# Long‐distance dispersal or postglacial contraction? Insights into disjunction between Himalaya–Hengduan Mountains and Taiwan in a cold‐adapted herbaceous genus, *Triplostegia*

**DOI:** 10.1002/ece3.3719

**Published:** 2017-12-20

**Authors:** Yan‐Ting Niu, Jian‐Fei Ye, Jin‐Long Zhang, Ji‐Zhong Wan, Tuo Yang, Xiao‐Xin Wei, Li‐Min Lu, Jian‐Hua Li, Zhi‐Duan Chen

**Affiliations:** ^1^ State Key Laboratory of Systematic and Evolutionary Botany Institute of Botany Chinese Academy of Sciences Beijing China; ^2^ University of Chinese Academy of Sciences Beijing China; ^3^ Beijing Botanical Garden Institute of Botany Chinese Academy of Sciences Beijing China; ^4^ Flora Conservation Department Kadoorie Farm and Botanic Garden Hong Kong SAR China; ^5^ School of Nature Conservation Beijing Forestry University Beijing China; ^6^ Sino‐African Joint Research Center Chinese Academy of Sciences Wuhan China; ^7^ Biology Department Hope College Holland MI USA

**Keywords:** conservation, Himalaya–Hengduan Mountain region, phylogeography, species distribution modeling, Taiwan, *Triplostegia*

## Abstract

Current disjunct patterns can result from long‐distance dispersal or postglacial contraction. We herein investigate the evolutionary history of *Triplostegia* to elucidate the disjunction between the Himalaya–Hengduan Mountain region (HHM) and Taiwan (TW). Genetic structure of *Triplostegia* was investigated for 48 populations using sequences from five chloroplast loci and the ribosomal nuclear internal transcribed spacer. Divergence time estimation, ancestral area reconstruction, and species distribution modeling (SDM) were employed to examine the biogeographic history of *Triplostegia*. Substantial genetic differentiation among populations from southwestern China (SW), Central China (CC), and TW was detected. *Triplostegia* was inferred to have originated in SW, and diversification began during the late Miocene; CC was colonized in the mid‐Pliocene, and TW was finally colonized in the early Pleistocene. SDM suggested an expansion of climatically suitable areas during the Last Glacial Maximum and range contraction during the Last interglacial in *Triplostegia*. Disjunction between HHM and TW in *Triplostegia* is most likely the consequence of topographic isolation and postglacial contraction. The potential climatic suitability areas for *Triplostegia* by 2070s (2061–2080) are predicted to slightly shrink and move northward. With continued global warming and human‐induced deforestation, extinction risk may increase for the cold‐adapted species, and appropriate strategies should be employed for ecosystem conservation.

## 1 INTRODUCTION

Organismal genetic structure and distributions have been greatly impacted by climatic oscillations, especially the Quaternary glacial/interglacial cycles (Comes & Kadereit, 1998; Hewitt, 2000; Lomolino, Riddle, Whittaker, & Brown, 2010). Compared with North America and northern Europe, East Asia was inferred to be less affected by glaciation except in the higher mountains (Pinot et al., 1999; Shi & Yao, 2002). Numerous species show disjunct distributions in the high mountains of East Asia (Li et al., 2016; Liu et al., 2014; Meng et al., 2017; Wang, 1989; Wang et al., 2013). In particular, disjunctions between the Himalaya–Hengduan Mountain region (HHM) and Taiwan (TW) have received extensive attention because of the geographically distant separation of the two regions by the Taiwan Strait and a terrestrial distance of over 2,000 km (Chen, Ying, & Lu, 2012; Wu & Wang, 2001; Ye, Chen, Liu, Qin, & Yang, 2012).

Himalaya–Hengduan Mountain region and TW are two of the most important biodiversity hotspots worldwide, with high species richness and endemism (Lu, Peng, Cheng, Hong, & Chiang, 2001; Myers, Mittermeier, Mittermeier, Da Fonseca, & Kent, 2000; Tang, Wang, Zheng, & Fang, 2006; Wei, Yang, Li, & Wang, 2010). HHM is located in southwestern China and includes the eastern Qinghai–Tibetan Plateau (QTP), Hengduan Mountains (Mts.), and adjacent areas with an average elevation of more than 4,000 m (Peng, Wan, & Luo, 2000; Zhang, Li, & Zheng, 2002). Dramatic climatic change events have occurred in HHM with the accelerated uplift of the QTP during the Cenozoic (Favre et al., 2015; Wen, Zhang, Nie, Zhong, & Sun, 2014). Taiwan is the largest subtropical mountainous island situated at the Tropic of Cancer in the monsoonal western Pacific region, which began to emerge during the late Miocene (*c*. 9–5 million years ago, Ma; Sibuet & Hsu, 1997, 2004). There are over 100 species pairs that show disjunct distributions between HHM and TW (Ye et al., 2012). Based on previous studies (Lu et al., 2001; Wang et al., 2013; Ye, Zhu, Chen, Zhang, & Bu, 2014), two hypotheses have been proposed to elucidate the formation of the HHM‐TW disjunction: long‐distance dispersal and postglacial contraction.

Long‐distance dispersal in plants usually involves rare events driven by dispersal vectors such as animals, currents, winds, or water (Nathan, 2006). Phylogeographic study of *Cunninghamia* revealed that populations in Taiwan were derived from continental Asia via long‐distance seed dispersal (Lu et al., 2001). The postglacial contraction hypothesis states that many species had a continuous distribution from Mainland China to TW during the Quaternary glacial period, and disjunction between HHM and TW occurred when taxa migrated to higher altitudes as the Earth became warmer (Chen et al., 2012; Wang, 1989; Wang et al., 2013; Ye et al., 2014). Most disjunct genera and species between HHM and TW are herbaceous (Chen, Ying, & Lu, 2012; Ye et al., 2012). However, few studies on the HHM‐TW disjunction focused on herbaceous plants, so it remains unclear whether similar mechanisms and timing shaped the disjunct patterns of woody and herbaceous plants between HHM and TW.

Recently, species distribution modeling (SDM) has been used to infer range shifts of plants and animals in response to the Quaternary climate oscillations (Jakob, Ihlow, & Blattner, 2007; Luo et al., 2016; Peterson, Ammann, & Diniz‐Filho, 2013). In general, most temperate species retracted into smaller refugial areas during glacial periods and would experience range expansion during interglacial periods (Bueno et al., 2016; Hewitt, 1996, 1999, 2000). However, several recent studies suggested a different biogeographic history for cold‐adapted coniferous species that inhabit high mountains, with range expansion during glacial periods and contraction during interglacial periods (Liu et al., 2013; Shao & Xiang, 2015; Tian, López‐Pujol, Wang, Ge, & Zhang, 2010; Wang et al., 2017). Compared with lowland species, mountainous species might be more likely to survive during climatic oscillations, because short‐distance vertical migrations allow them to track their optimal temperature niche (Hoorn, Mosbrugger, Mulch, & Antonelli, 2013; Sandel et al., 2011). Nevertheless, continued global warming in the near future may be a major threat to cold‐adapted species, especially those that are already confined to mountaintops or islands, because migration to higher elevations may be not possible or range shifts are not fast enough to track suitable climate (Wiens, 2016). How organisms responded to the Quaternary climatic oscillations and predictions of response to future climate can likely be inferred with short‐lived herbaceous plants, which have far more life cycles within a given time period and thus may respond more quickly to rapidly changing environments (Comes & Kadereit, 1998; Faye et al., 2016; Zanne et al., 2014).

As an herbaceous genus of Caprifoliaceae *s.l*. (The Angiosperm Phylogeny Group, 2016), *Triplostegia* Wall. ex DC. has a disjunct distribution between HHM and TW (see Figure S1), with only a few sporadic records in Indonesia (Hong, Ma, & Barrie, 2011; Zhang, Chen, Li, Chen, & Tang, 2003). *Triplostegia* is sensitive to habitat and climatic changes and requires a specific forest habitat that has fertile soil and an altitude ranging from 1,500 to 4,000 m; these features make this genus an ideal model for studying formation of HHM‐TW disjunctions and how herbs respond to Quaternary climate oscillations and future global warming. By sequencing five chloroplast DNA (cpDNA) regions and the ribosomal nuclear internal transcribed spacer (ITS) for 397 individuals from 48 populations, estimating divergence time, reconstructing ancestral areas, and conducting SDM, we (1) investigated the genetic structure and identified possible refugia for *Triplostegia*; (2) reconstructed the biogeographic history of *Triplostegia* and explored how the HHM‐TW disjunction formed; and (3) predicted the future climatically suitable areas of the cold‐adapted *Triplostegia* under a global warming scenario.

## 2 MATERIALS AND METHODS

### 2.1 Population sampling

*Triplostegia* includes two species: *T. glandulifera* Wall. ex DC. and *T. grandiflora* Gagnep. (Peng, Tobe, & Takahashi, 1995; Pyck & Smets, 2004; Zhang, Chen, Li, Chen, & Tang, 2003). *Triplostegia grandiflora* is endemic to China (confined to Yunnan and Sichuan Provinces), and its geographical range overlaps with that of *T. glandulifera* (Hong, Ma, & Barrie, 2011). Both species are perennial herbs only with minor differences in flower size. We herein treated *Triplostegia* as a monotypic genus based on the evidence from pollen morphology and phylogenomic analysis of nine complete chloroplast genomes (Niu et al., unpublished).

From September 2011 to August 2014, we collected 397 individuals from 48 natural populations, which covered the entire geographical range of *Triplostegia* in China. Generally, 10–12 individuals were arbitrarily selected from a population, whereas three to six individuals were collected from populations with few individuals (Table S1). For each individual, leaves were collected and dried in silica gel for DNA extraction. Voucher specimens were deposited at the Herbarium of Institute of Botany, Chinese Academy of Sciences (PE).

### 2.2 DNA extraction, amplification, and sequencing

Total genomic DNA was extracted from silica gel‐dried leaves using a plant total genomic DNA kit following the manufacturer's protocol (Biomed, Beijing, China). Five cpDNA regions (*psb*K–*psb*I, *rpl*20–*rps*12, *trn*H–*psb*A, *trn*L–F, and *trn*S–*trn*G) and ITS were amplified with primers described in Baldwin (1993), Hamilton (1999), Lahaye, Savolainen, Duthoit, Maurin, and Van der Bank (2008), Sang, Crawford, and Stuessy (1997), Soejima and Wen (2006), Taberlet, Gielly, Pautou, and Bouvet (1991), and White, Bruns, Lee, and Taylor (1990). PCR primers and amplification profiles for each DNA region are shown in Table S2. The PCR products were purified and sequenced by Beijing Biomed Gene Technique Company (http://www.biomed168.com/goods). The forward and reverse sequences were assembled in GENEIOUS 9.1.4 (http://www.geneious.com; Kearse et al., 2012). Sequences were initially aligned using MAFFT 1.3.5 (Katoh, Misawa, Kuma, & Miyata, 2002) and then manually adjusted in GENEIOUS.

### 2.3 Genetic structure analyses

The number of haplotypes, haplotype diversity (*H*_d_), and nucleotide diversity (π) were calculated in DNASP 5.0.0 (Librado & Rozas, 2009). All haplotype sequences identified in this study were deposited in GenBank (https://www.ncbi.nlm.nih.gov/; see Table S3 for accession numbers). Genealogical relationships among haplotypes were investigated using the median‐joining network method as implemented in NETWORK 5.0.0 (Bandelt, Forster, & Röhl, 1999).

The average gene diversity within populations (*H*_S_), total gene diversity (*H*_T_), and the coefficients of differentiation (*G*_ST_, a population differentiation estimate based solely on haplotype frequencies, and *N*_ST,_ a parameter that considers both haplotype frequencies and their genetic divergence) were estimated using PERMUT 1.0.0 (Pons & Petit, 1996) with 1,000 random permutations. When *N*_ST_ is larger than *G*_ST_, closely related haplotypes occur more often in the same area than less closely related haplotypes, which indicates strong phylogeographic structure.

Spatial genetic structure of cpDNA and ITS haplotypes was analyzed by SAMOVA 1.0 (Dupanloup, Schneider, & Excoffier, 2002). This program uses a simulated annealing approach to identify groups of populations (*K*) that are geographically homogeneous and maximally differentiated from each other. To maximize the proportion of total genetic variance observed between groups (*Φ*_CT_), 1,000 iterations were run for *K *= (2, …, 8) from each of 100 random initial conditions. To further quantify genetic differentiation among populations and groups, the hierarchical analysis of molecular variance (AMOVA) was estimated in ARLEQUIN 3.5.1 (Excoffier & Lischer, 2010) with significance tests based on 1,000 permutations.

### 2.4 Demographic reconstruction

Mismatch distribution analysis with a spatial expansion model (Rogers & Harpending, 1992) was conducted in ARLEQUIN to infer the historical demography of *Triplostegia* based on both cpDNA and ITS datasets. The goodness of fit based on the sum of squared deviation (SSD) and Harpending's raggedness index (*H*_Rag_) was tested using 1,000 parametric bootstrap replicates. If the expansion model was not rejected, the formula *t *= τ/2*u* was used to estimate the expansion time (*t*), where *u *= μ*kg* [μ, substitution rate, substitutions per site per year, subs/site/year; *k*, average sequence length of cpDNA; *g*, generation time in years (i.e., age of first reproduction; approximated as 1 year, J. F. Ye, personal observation)]. In addition, statistical significance of population expansion was further examined with two neutrality tests, Tajima's *D* (Tajima, 1989) and Fu's *F*_S_ (Fu, 1997), as implemented in DNASP, in which significantly negative values indicate population expansion.

### 2.5 Phylogenetic relationships among haplotypes

Phylogenetic analyses for cpDNA and ITS haplotypes were performed using the maximum likelihood (ML), Bayesian inference (BI), and neighbor‐joining (NJ) methods, with gaps treated as missing data. The ML analysis was conducted using RAxML 8.2.8 (Stamatakis, 2006, 2014) using a GTR + G substitution model with 1,000 bootstrap (BS) replicates (Stamatakis, Hoover, & Rougemont, 2008) on the CIPRES Science Gateway Portal (https://www.phylo.org/portal2/; Miller, Pfeiffer, & Schwartz, 2010). The best‐fitting model of nucleotide substitutions was selected in JMODELTEST 2.1.4 (Darriba, Taboada, Doallo, & Posada, 2012) under the Akaike information criterion (AIC). BI analyses were conducted in MRBAYES 3.2 (Ronquist & Huelsenbeck, 2003; Ronquist et al., 2012) with the best‐fit model determined above. Two Markov chain Monte Carlo (MCMC) chains were run for two million generations with one tree sampled every 1,000 generations. The average standard deviation of the split frequencies approached 0.01, which indicated that two runs converged to a stationary distribution. After discarding the first 25% trees as burn‐in, a 50% majority‐rule consensus tree was constructed from the remaining trees to estimate posterior probabilities (PP). NJ analysis for ITS was carried out in MEGA 6.0.0 (Tamura, Stecher, Peterson, Filipski, & Kumar, 2013) with 1,000 BS replicates.

### 2.6 Divergence time estimation

To estimate the crown age of *Triplostegia*, we downloaded sequences of three cpDNA regions (*trn*H–*psb*A, *trn*L–F, and *trn*S–*trn*G) for 41 Dipsacales species (including five Adoxaceae species as out‐groups) from GenBank (Table S4) that represent all major lineages of Dipsacales (Bell & Donoghue, 2005; Carlson, Linder, & Donoghue, 2012; Wang, Landrein et al., 2015). Sequences were assembled, with 16 *Triplostegia* haplotypes identified from the same three cpDNA regions, to represent all 20 cpDNA haplotypes identified in the phylogeographic analyses. Bayesian searches for tree topologies and node ages of this cpDNA dataset were conducted in BEAST 1.7.3 (Drummond & Rambaut, 2007), which was run on the CIPRES Science Gateway Portal. An uncorrelated lognormal relaxed clock (Drummond, Nicholls, Rodrigo, & Solomon, 2002) was applied with the GTR + G substitution model and a Yule process tree prior. A secondary calibration point (52.8–89.0 Ma; Magallón, Gómez‐Acevedo, Sánchez‐Reyes, & Hernández‐Hernández, 2015) was used to constrain the crown group of Dipsacales that assumed a normal distribution with a mean (± *SD*) of 70.94 ± 11 Ma (node 1 in Figure S2).

Three fossil dates were used to assign minimum age constraints on three internal nodes of Dipsacales with a lognormal distribution (nodes 2–4 in Figure S2). The earliest fossil record of *Viburnum* from the late Paleocene to early Eocene (*c*. 56 Ma; Baskin et al., 2006; Wing et al., 1995) was used to constrain the crown group of Adoxaceae (node 2 in Figure S2; mean = 0, *SD* = 1.0, offset = 56 Ma) following Moore and Donoghue (2007). Fossil seeds of *Weigela* have been reported from the Miocene and Pliocene in Poland (Łańcucka‐Środoniowa, 1967), the Miocene in Mammoth Mts., Eastern Russia, the Oligocene and Miocene of Western Siberia (Dorofeev, 1963), and the Miocene in Denmark (Friis, 1985). We thus constrained divergence between *Weigela* and its sister *Diervilla* to 23 Ma (node 3 in Figure S2; mean = 0, *SD* = 1.0, offset = 23 Ma) following Wang, Landrein et al. (2015). Finally, we used the fossil fruits of *Diplodipelta* from the late Eocene Florissant flora of Colorado (36–35 Ma; Manchester & Donoghue, 1995) to constrain the stem age of *Dipelta* (node 4 in Figure S2; mean = 0, *SD* = 1.0, offset = 36 Ma) following Bell and Donoghue (2005) and Wang, Landrein et al. (2015). We assessed estimate robustness by sequentially removing each calibration while retaining the other three calibrations (Wahlberg, Wheat, & Peña, 2013).

To determine the intraspecific node ages within *Triplostegia*, we applied a similar BEAST analysis to a dataset that included all 20 haplotypes of five cpDNA regions, with *Dipsacus asper* as the out‐group. We used the median stem age and crown age of *Triplostegia* obtained from the Dipsacales chronogram to constrain the root and crown age of *Triplostegia*, respectively (nodes A and B in Figure S2). The normal distribution and a constant‐size coalescent tree prior were applied. MCMC runs were performed, each of 10^8^ generations, with sampling every 10^4^ generations, following a burn‐in of the initial 10% of cycles. We used TRACER 1.5.1 (Raymond & Rousset, 2009) to examine the stationarity of runs. Trees were then compiled into a maximum clade credibility tree using TREEANNOTATOR 1.7.5 (Drummond, Suchard, Xie, & Rambaut, 2012). FIGTREE 1.4.0 (Rambaut, 2009) was used to display mean ages and highest posterior density intervals at 95% for each node.

### 2.7 Ancestral area reconstruction

We used the Bayesian Binary MCMC (BBM) method implemented in RASP 3.0.0 to estimate the possible ancestral areas of *Triplostegia* with the trees obtained from the intraspecific BEAST analysis (Yu, Harris, Blair, & He, 2015). Three geographical regions that represent the current distribution of *Triplostegia* were defined: southwestern China (SW; East Himalayas and Hengduan Mts.); Central China (CC; surrounding areas of the Sichuan Basin); and Taiwan (TW; Taiwan mountain ranges). SW and CC were defined as two separate regions based on their unique haplotypes and altitudinal gradients, despite their geographical proximity. *Triplostegia* distribution was inferred based on the specimen information from the Chinese Virtual Herbarium (CVH, http://www.cvh.org.cn/), Global Biodiversity Information Facility (GBIF, http://www.gbif.org/), and Specimens Database of Native Plants in Taiwan (SDNPT, http://www.hast.biodiv.tw/Announce/newsC.aspx). The out‐group (*Dipsacus asper*) is found in both SW and CC, so we set its distribution as SW/CC. The number of maximum areas at each node was set to three. To account for phylogenetic uncertainty, 1,000 trees from BEAST were randomly chosen for BBM analysis. We set the root distribution to null, applied 10 MCMC chains with the JC + G model, and sampled the posterior distribution every 100 generations for 10^6^ generations.

### 2.8 Species distribution modeling

We used MaxEnt species distribution modeling (henceafter MaxEnt) to predict past, current, and future climatically suitable areas for *Triplostegia* (Phillips, Anderson, Dudík, Schapire, & Blair, 2017). MaxEnt is one of the most popular tools to model species distributions based on occurrence records and relevant climatic variables, and it was conceptualized in Merow, Smith, and Silander (2013) and Phillips, Anderson, and Schapire (2006) for details.

We first downloaded 19 bioclimatic variables with the 2.5 arc‐minute spatial resolution available from database of WorldClim 1.4 (http://www.worldclim.org; Hijmans, Cameron, Parra, Jones, & Jarvis, 2005). To eliminate multicollinearity effects in the parameter estimates of SDM, we excluded variables with |Pearson's *R*| ≥ .85, and selected nine variables as climatic predictors to model the past, current, and future climatically suitable areas of *Triplostegia* (Camargo, Werneck, Morando, Sites, & Avila, 2013). The resultant climatic variables included annual mean temperature (Bio1; °C), mean diurnal range (Bio2; °C), isothermality (Bio3; dimensionless), temperature seasonality (Bio4; Standard Deviation*100), max temperature of warmest month (Bio5; °C), mean temperature of coldest month (Bio6; °C), mean temperature of driest quarter (Bio9; °C), annual precipitation (Bio12; mm), and precipitation seasonality (Bio15; Coefficient of Variation). These nine climatic variables (i.e., the maximum, minimum, mean and standard deviation of temperature and precipitation) can influence the distribution and physiological performance of plant species. Altitude is an important factor to predict suitable habitats for *Triplostegia*, but we did not take it into consideration due to its close relationship with temperature and precipitation.

Current distribution of *Triplostegia* was developed using the same nine bioclimatic variables for current climate (1960–1990). To model the paleoclimatic distribution of *Triplostegia*, we used three global climate models (GCMs: CCSM4, MIROC‐ESM, and MPI‐ESM‐P) for the Last Glacial Maximum (LGM; 0.021 Ma), but only considered NCAR‐CCSM for the Last interglacial (LIG; 0.12–0.14 Ma) because it is the sole GCM available in the database of WorldClim. To predict the future climatically suitable areas of *Triplostegia* in 2050s (average for 2041–2060) and 2070s (average for 2061–2080), we used three GCMs (CCSM4, MIROC‐ESM and MPI‐ESM‐LR) and two greenhouse gas concentration scenarios as representative concentration pathways (RCPs) of 4.5 (mean, 780 ppm; range, 595–1,005 by 2100) and 8.5 (mean, 1,685 ppm; range, 1,415–1,910 by 2100), representing the low and high gas concentration scenarios, respectively (http://www.ipcc.ch/; http://www.ccafs-climate.org/). RCP 8.5 may have larger cumulative concentrations of carbon dioxide than RCP 4.5, resulting in a different pattern of future climate change in response to various anthropogenic concentrations of greenhouse gases and other pollutants (http://www.ipcc.ch/report/ar5/).

We corrected all the occurrence data to reduce the negative effect of sampling bias on the performance of the SDMs. For records from GBIF, CVH, and SDNPT, we excluded the duplicated entries within a 2.5 arc‐minute pixel (4.3 km at the equator) and omitted entries with implausible geographical coordinates using ArcGIS 10.2 (ESRI Inc., Redlands, CA, USA; http://www.esri.com/software/arcgis/arcgis-for-desktop) and Google Earth (http://earth.google.com/; Gueta & Carmel, 2016; Maldonado et al., 2015). We also checked voucher photos for records from CVH and SDNPT to make sure that the species are correctly identified. Finally, a total of 250 cleaned occurrence records were obtained from GBIF, CVH, SDNPT, and our own collections (Table S1 and Figure S3), which well covered the distribution range of *Triplostegia* (Hong, Ma, & Barrie, 2011).

As suggested by previous studies (Halvorsen et al., 2016; Martinez‐Freiria, Velo‐Antón, & Brito, 2015; Merow, Smith, & Silander, 2013; Phillips et al., 2017; Radosavljevic, Anderson, & Araújo, 2014), our modeling was set as follows: (1) the regularization multiplier level was at two to produce smooth and general response curves that represent a biologically realistic model; (2) cloglog output format was used to evaluate distribution probability of *Triplostegia*; (3) ten independent replicates of cross‐validation procedures for model testing; (4) the maximum number of background points was set to 10^4^; and (5) jackknife analyses of the regularized gain using training data were conducted to elucidate the importance of each predictor (Fawcett, 2006). The other settings followed the suggestions of Merow, Smith, and Silander (2013). No obvious differences were detected among the predictions based on the three GCMs (Figure S4). To decrease the prediction uncertainty, mean consensus for the pixels of distribution probabilities modeled by three GCMs was calculated to assess the distributions of *Triplostegia* during LGM and the future (RCPs 4.5 and 8.5 for both 2050s and 2070s).

We evaluated the model performance by calculating the area under the receiver operating characteristic (ROC) curve (AUC). AUC was used to assess the accuracy of predictions, and models with AUC values larger than 0.7 were considered useful for our study (Phillips & Dudík, 2008). However, using the traditional AUC may be not sufficient to assess the performance of SDMs. We herein used a binomial test (based on the training omission rate) for model validation based on the occurrence data only. The training omission rate is the proportion of training occurrence records among pixels of predicted absences (Phillips & Dudík, 2008; Phillips, Anderson, & Schapire, 2006). One‐sided test was used for the null hypothesis, and the test points are predicted no better than those by a random prediction (Phillips & Dudík, 2008; Phillips, Anderson, & Schapire, 2006). We used the 11 common thresholds defaulted by MaxEnt to define binomial probabilities (Phillips, Anderson, & Schapire, 2006). The models with <17% training omission rate were useful for our study (Phillips, Anderson, & Schapire, 2006).

## 3 RESULTS

### 3.1 Genetic diversity and population differentiation

**Figure 1:**
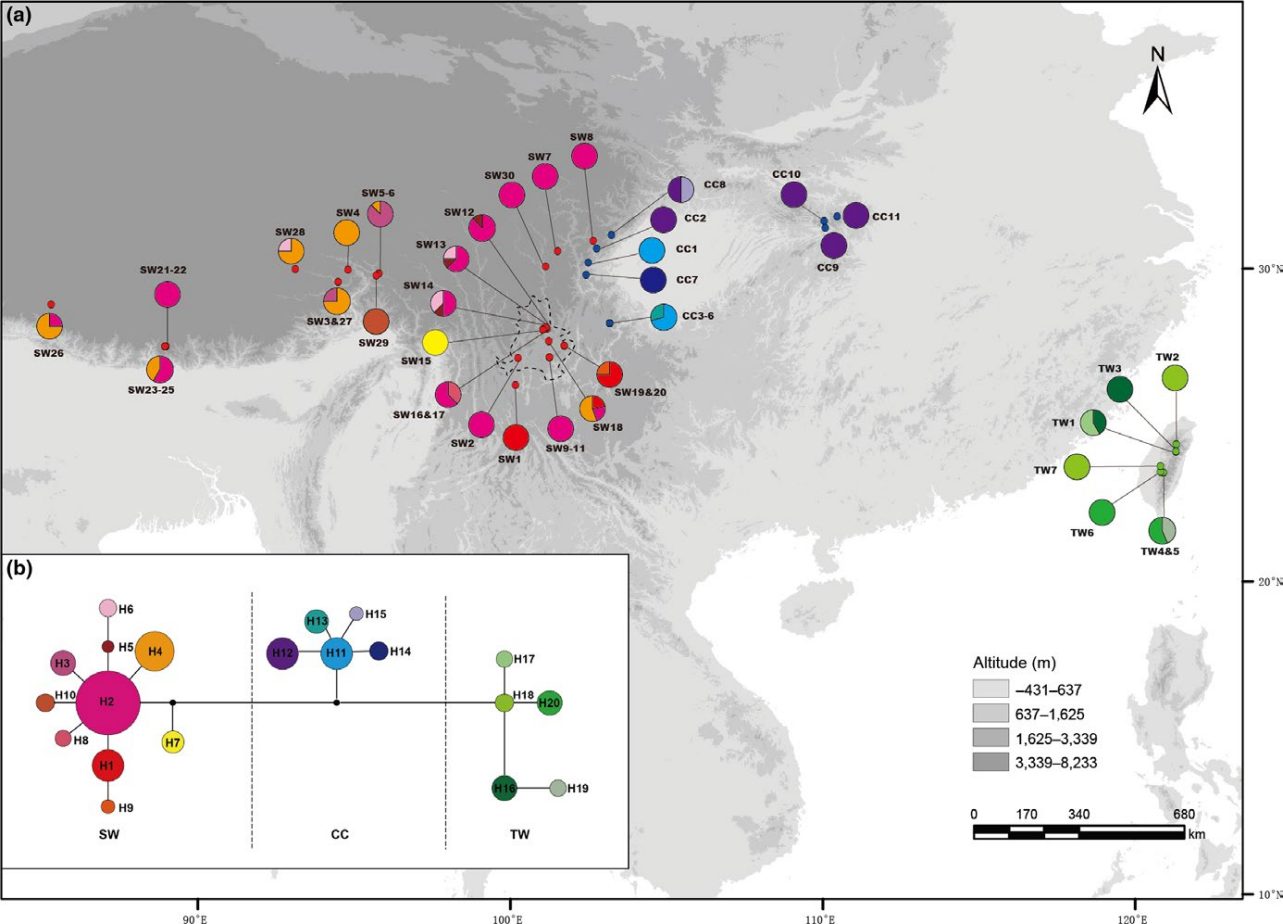
(a) Geographical distribution of the cpDNA haplotypes detected in *Triplostegia* (see Table S1 for population codes) and (b) network of genealogical relationships among 20 haplotypes. Each circle represents a single haplotype with sizes in proportion to frequency. Black dots in network represent missing haplotypes. Potential interglacial refugia recognized by this study are encircled by dashed lines

We obtained 2,812 bp of cpDNA sequences with 430 bp of *psb*K–*psb*I, 718 bp of *rpl*20–*rps*12, 201 bp of *trn*H–*psb*A, 788 bp of *trn*L–F, and 675 bp of *trn*S–*trn*G. There were 20 haplotypes defined by 33 polymorphic sites (Table S1) and three haplotype clusters that corresponded to three geographical regions: SW, CC, and TW (Figure 1a). *H*_d_ and π for the cpDNA dataset were estimated to be 0.87 and 0.24 × 10^−2^, respectively (Table S1). *H*_T_ and *H*_S_ were 0.88 and 0.18, respectively. The coefficients of differentiation measured over the 48 populations were *G*_ST_ = 0.80 and *N*_ST_ = 0.96. Permutation test revealed that *N*_ST_ was significantly higher than *G*_ST_ (*p *<* *.05), which indicates strong phylogeographic structure for the *Triplostegia* cpDNA haplotypes. The highest levels of cpDNA sequence diversity (π) were recorded within the SW group and the lowest levels in the CC group (Table S1). Differences in cpDNA sequences were highly significant among the three geographical regions (*F*_CT_ = 0.85, *p *<* *.001, AMOVA; Table 1), populations within regions (*F*_SC_ = 0.77, *p *<* *.001, AMOVA; Table 1), and among all populations (*F*_ST_ = 0.97, *p *<* *.001, AMOVA; Table 1). Differences in cpDNA sequences were significant between every two of the three geographical regions (Table S5). The highest differentiation index was found between SW and TW (*F*_CT_ = 0.88^***^), and the lowest between CC and TW (*F*_CT_ = 0.68^***^).

**Table 1:** Molecular variances of cpDNA and ITS for 48 *Triplostegia* populations

Gene type	Source of variation	*df*	SS	VC	PV	Fixation index
cpDNA	Among regions	2	1,167.64	5.46	85.21	*F*_CT_ = 0.85***
	Among populations	45	280.45	0.73	11.38	*F*_SC_ = 0.77***
	Within populations	349	76.29	0.22	3.14	*F*_ST_ = 0.97***
	Total	396	1,524.38	6.40		
ITS	Among regions	2	1,070.74	5.01	88.48	*F*_CT_ = 0.89***
	Among populations	45	237.82	0.64	11.29	*F*_SC_ = 0.98***
	Within populations	349	4.64	0.013	0.23	*F*_ST_ = 0.99***
	Total	396	1,313.20	5.66		

*df*, degree of freedom; SS, sum of squares; VC, variance components; PV, percentage of variation; ***, *p *<* *.001, 1,000 permutations.

**Figure 2:**
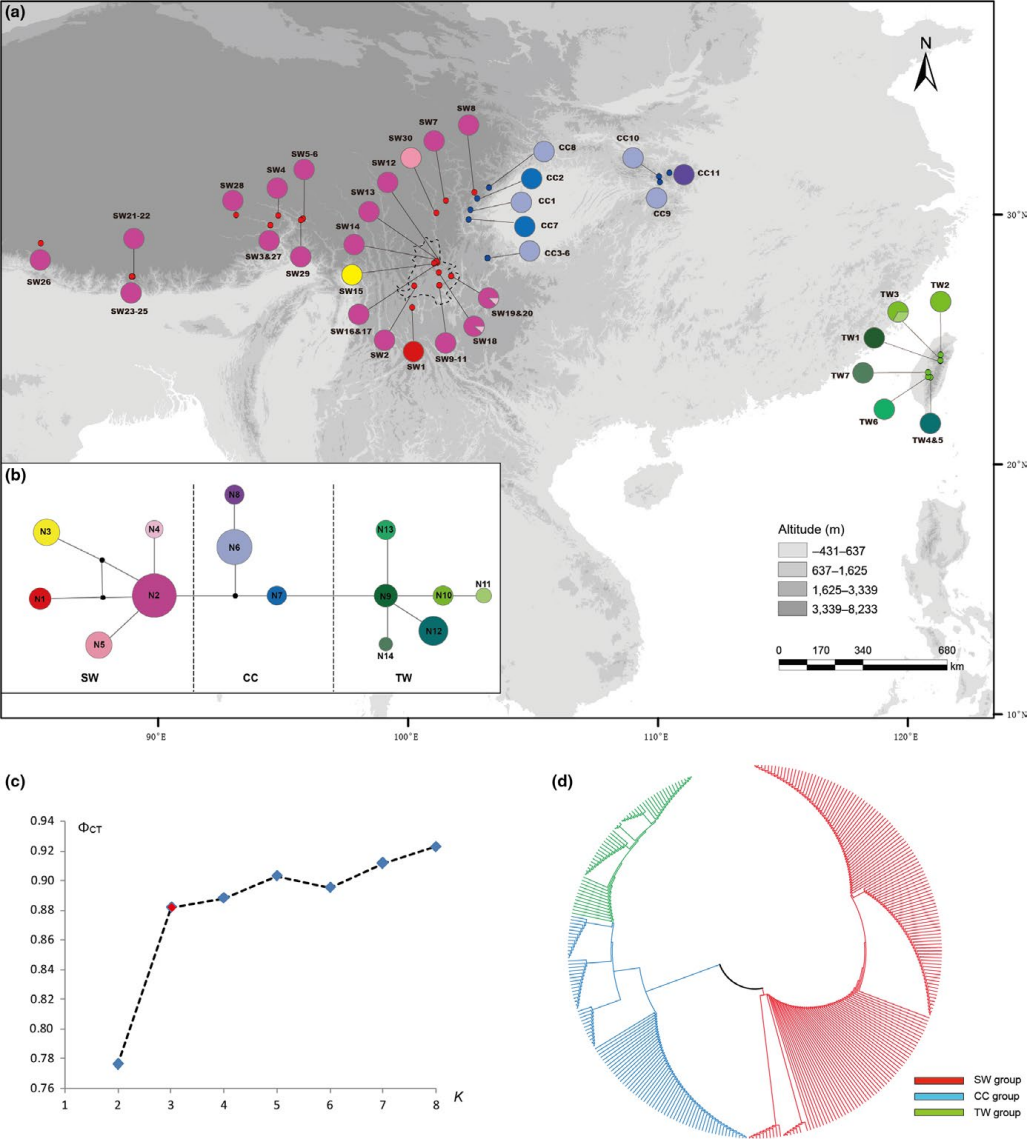
Analysis based on ITS dataset. (a) Geographical distribution of haplotypes in *Triplostegia* (see Table S1 for population codes); (b) network of genealogical relationships between 14 haplotypes. Each circle represents a single haplotype sized in proportion to its frequency. Black dots in the network represent missing haplotypes; (c) results of SAMOVA (the red point indicated the optimized number of groups *K*); and (d) neighbor‐joining tree without out‐group. Potential interglacial refugia recognized by this study are encircled by dashed lines

The matrix for aligned ITS had 573 bp with 36 polymorphic sites and 14 ITS haplotypes (see Table S3 for accession numbers). *H*_d_ and π for ITS were estimated to be 0.67 and 0.011, respectively (Table S1). Network and geographical distribution analysis based on ITS also recognized three groups in SW, CC, and TW (Figure 2). The highest level of ITS π was recorded within the SW group and the lowest in the CC group (Table S1). Differences in ITS sequences were highly significant among the three geographical regions (*F*_CT_ = 0.89, *p *<* *.001, AMOVA; Table 1), between populations within regions (*F*_SC_ = 0.98, *p *<* *.001), and among all populations (*F*_ST_ = 0.99, *p *<* *.001). Differences in ITS sequences were significant between each pair of geographical regions (Table S6). The highest differentiation index was observed between SW and TW (*F*_CT_ = 0.91^***^) and the lowest between CC and TW (*F*_CT_ = 0.84^***^). Analysis of the ITS variation across all populations revealed that the total gene diversity (*H*_T_ = 0.67) was much higher than the within‐population diversity (*H*_S_ = 0.025). The coefficients of differentiation measured over the 48 populations were *G*_ST_ = 0.96 and *N*_ST_ = 0.99. Permutation test revealed that *N*_ST_ was significantly higher than *G*_ST_ (*p *<* *.05), which indicated a strong phylogeographic structure for ITS haplotypes. SAMOVA indicated that values of *Φ*_CT_ reached a plateau at 0.88 for three groups (*K *=* *3; Figure 2c), which corresponded to the three clades in SW, CC, and TW.

### 3.2 Phylogenetic relationships and divergence times

Both ML and BI analyses based on the 20 cpDNA haplotypes supported three lineages within *Triplostegia* that corresponded to their distributions in SW, CC, and TW (Figure S5). However, incongruence existed regarding the relationships among three lineages between the BI (SW(CC, TW)) and ML (TW(SW,CC)) analyses, although the support values were not high. The ML, BI, and NJ analyses based on ITS retrieved the same topology (SW(CC, TW)) as the BI tree based on cpDNA (Figure S5).

The chronogram for Dipsacales derived from cpDNA supported the monophyly of the 16 *Triplostegia* haplotypes (PP = 1.00; Figure S2). The stem age of *Triplostegia* was estimated to be *c*. 48.29 Ma (95% highest posterior density, HPD: 31.65–66.45 Ma; node A; Table S7) and crown age of *Triplostegia* as *c*. 10.92 Ma (95% HPD: 4.28–23.76 Ma; node B; Table S7). The divergence time within *Triplostegia* showed that the coalescent time of all 20 haplotypes was during the late Miocene (6.79 Ma, 95% HPD: 2.50–13.6 Ma; node I in Figure S5). Within the CC–TW lineage, CC and TW group split *c*. 4.03 Ma (95% HPD: 1.31–8.62 Ma; node II in Figure S5). Split within the three groups occurred during the early Pleistocene: SW (*c*. 1.98 Ma, 95% HPD: 0.62–4.83 Ma; node III in Figure S5), CC (*c*. 1.41 Ma, 95% HPD: 0.26–3.85 Ma; node IV in Figure S5), and TW (*c*. 2.15 Ma, 95% HPD: 0.48–5.32 Ma; node V in Figure S5). For this chronogram, BEAST provided an average substitution rate of 0.49 × 10^−9^ subs/site/year, which is similar to that of *Tetrastigma hemsleyanum* derived from three cpDNA markers (*pet*L–*psb*E, *trn*K–*mat*K, and *rbc*L; 0.50 × 10^−9^ subs/site/year; Wang, Jiang et al., 2015).

### 3.3 Demographical analyses

**Figure 3:**
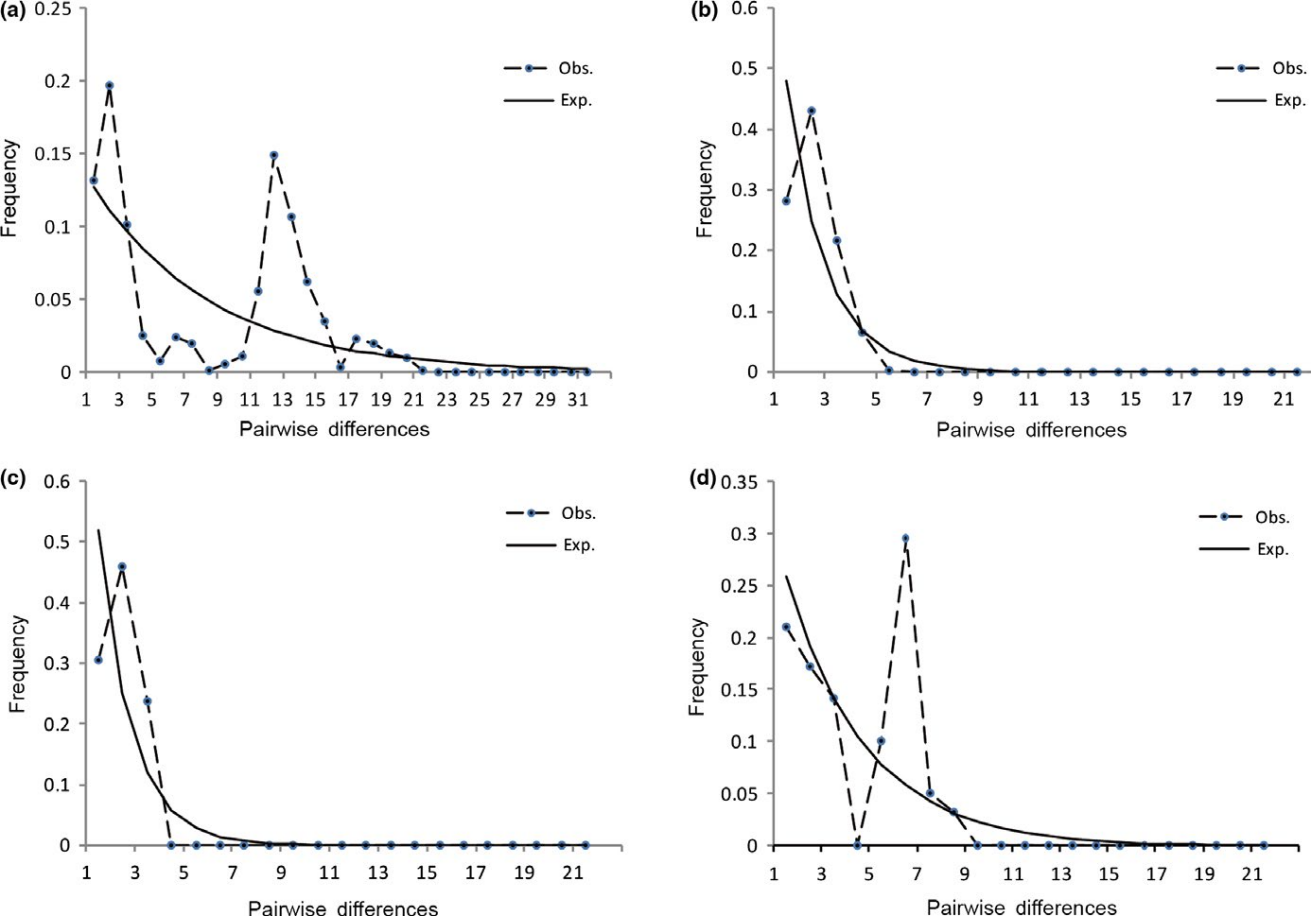
Number of pairwise nucleotide differences in *Triplostegia* based on chloroplast DNA sequences in all areas (a), southwestern China (b), Central China (c), and Taiwan (d). Black dots and dashed line show observed values (Obs.); solid lines indicate expected values (Exp.) under a model of sudden (stepwise) population expansion

The overall observed mismatch distribution of pairwise nucleotide differences was not unimodal and rejected the hypothesis that there was recent demographic population growth in *Triplostegia* (Figures 3a and S6). However, for each subregion (SW, CC, and TW), populations had a unimodal shape in the mismatch distribution. This supported the hypothesis that there had been recent demographic population expansion in the genus (Figures 3b–d and S6b–d). However, based on the SSD and *H*_Rag_ statistics of cpDNA sequences (Table 2), only SW had a good statistical support for the spatial expansion model (*p *>* *.05). In addition, Tajima's *D* and Fu's *F*_S_ values of cpDNA and ITS sequences were all negative in SW, which further indicated that populations in SW experienced sudden expansion (Table 2 & Figure S6). Based on the value of τ and assuming a substitution rate of 0.49 × 10^−9^ subs/site/year for cpDNA, the demographic expansion in SW populations was inferred to have occurred *c*. 0.33 Ma (Table 2).

**Table 2:** Results of mismatch distribution analysis and neutrality tests for pooled populations of *Triplostegia* lineages

Region	τ	θ_0_	θ_1_	SSD (*p* value)	*H*_Rag_ (*p* value)	*D*	*F*_S_	*t*
Southwestern China	0.94	0.11	30,000.62	.16	.30	−0.79	−2.38	0.33
Central China	0.63	0.08	0.60	.013*	.027*	0.38	0.22	—
Taiwan	1.53	0.00	14,285.81	.031*	.16	2.26*	3.93	—

τ = 2*ut*, where *t* is the expansion time and *u* is the mutation rate per generation; θ_0_, pre‐expansion population size; θ_1_, postexpansion population size; SSD, sum of squared deviations; *H*_Rag_, Harpending's raggedness index; *t*, time since expansion (Ma); *, *p *<* *.05.

### 3.4 Ancestral area reconstruction

**Figure 4:**
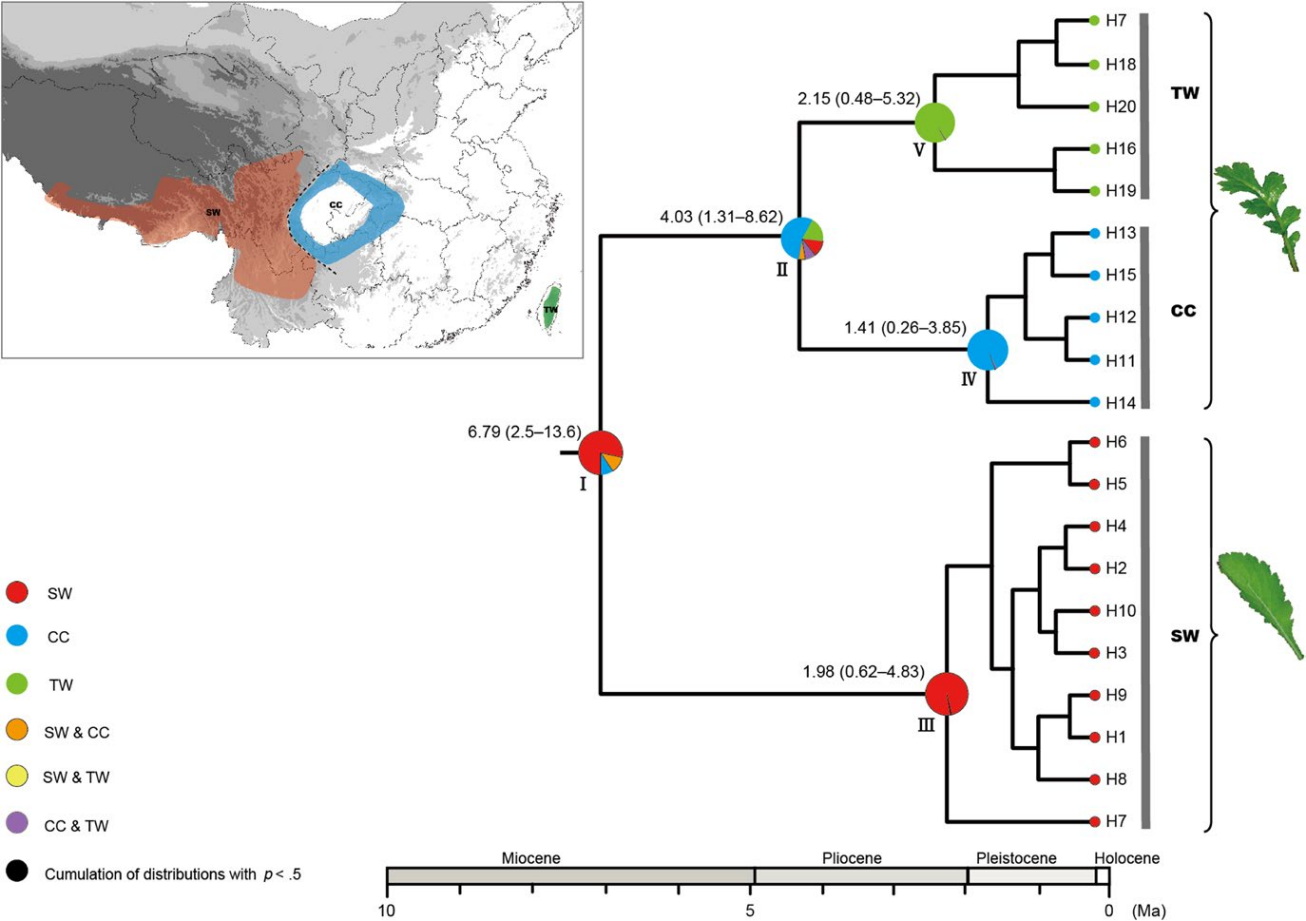
Ancestral area reconstructions based on the Bayesian binary Markov chain Monte Carlo (BBM) method implemented in RASP using the BEAST‐derived chronogram of *Triplostegia*. The upper left map shows major distribution divisions of *Triplostegia* in China: southwestern (SW), Central (CC), and Taiwan (TW). Pie chart at each node illustrates the marginal probabilities for each alternative ancestral area derived from BBM with the maximum area number set to three. The legend denotes possible ancestral ranges at different nodes. Ages of key nodes are labeled above branches. The out‐group (*Dipsacus asper*, see Figure S5) was not shown here

Based on the topology of the intraspecific chronogram (Figure S5), BBM analysis supported an ancestral distribution in SW (node I in Figure 4). BBM analysis inferred that *Triplostegia* colonized CC in the mid‐Pliocene (*c*. 4.03 Ma; 95% HPD: 1.30–8.60 Ma; node II in Figures 4 and S5) and arrived TW in the early Pleistocene (*c*. 1.98 Ma, 95% HPD: 0.62–4.83 Ma; node III in Figures 4 and S5). The three clades, which corresponded to their distributions in SW, CC, and TW, each had their own most recent common ancestors (nodes III, IV, and V in Figure 4), which indicated possible *in situ* diversification in the three regions.

### 3.5 Species distribution modeling

The training and test AUC values of the models were larger than 0.9, and all the training omission rates were <17%, indicating a good performance of the predictive model. The jackknife results showed that temperature was the most influential factor.

**Figure 5:**
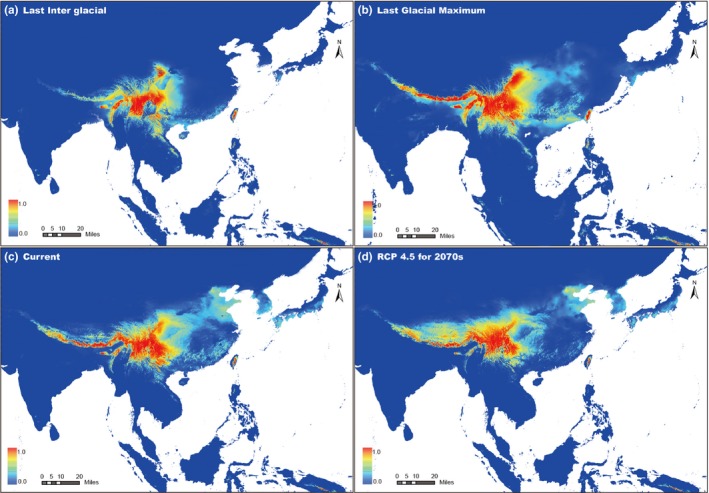
Species distribution models showing climatic suitability for *Triplostegia* in East Asia: (a) the Last interglacial (LIG; 0.12–0.14 Ma); (b) the Last Glacial Maximum (LGM; 0.021 Ma); (c) current conditions (current, 1960–1990); and (d) 2070s (2061–2080) with the representative concentration pathways (RCP) of 4.5. Climatic suitability increases with color from blue to red. Resolution for the potential distribution map is 2.5 arc‐minutes

During the LIG, high climatic suitability areas for *Triplostegia* were more restricted than its current range in southwestern China (e.g., the eastern Himalayas, Hengduan mountain regions; Figure 5a). The LGM modeling predicted a more widespread of high climatic suitability areas than current in East China, extending southward into Taiwan, plus a higher suitability of HHM regions (Figure 5b). The predicted suitable habitats of *Triplostegia* under current conditions were generally similar to the actual species distribution (Figure 5c). High climatic suitability areas for *Triplostegia* by 2050s (Figure S7a,b) and 2070s (Figures 5d and S7c) may experience northward range shift in Mainland China, and the suitability slightly decline in southern Hengduan Mountain regions and Taiwan.

## 4 DISCUSSION

### 4.1 Genetic diversity and potential refugia

Results from PERMUT (*N*_ST_ > *G*_ST_, *p *<* *.05), AMOVA (Table 1), and SAMOVA (Figure 2c) revealed strong genetic structure for the *Triplostegia* populations from SW, CC, and TW (Figures 1 and 2). The three distinct haplotype groups could be distinguished by a strong underlying phylogenetic hierarchy that supports three major clades in *Triplostegia* that correspond to their distributions in SW, CC, and TW (Figures 2d and S5). The strong genetic structure among *Triplostegia* populations is consistent with the three great topographic steps in China. SW populations are all from the first‐step terrain in China with an average elevation above 3,144 m, and have 10 cpDNA and five ITS haplotypes (Table S1). CC populations are distributed on the second‐step terrain around Sichuan Basin (average elevation: 2,238 m), and share five cpDNA and three ITS haplotypes. TW populations are all from the central mountain range of Taiwan (average elevation: 2,321 m) and possess five cpDNA and six ITS haplotypes. Strong geography‐haplotype‐correlated genetic structures were also detected in birds (Qu et al., 2015; Wang et al., 2013), insects (Ye et al., 2014), and plants (Gao et al., 2007; Meng et al., 2017), which highlight the importance of topographic complexity in promoting species differentiation both by increasing habitat diversity and limiting gene flow between elevation‐restricted populations (Hoorn, Mosbrugger, Mulch, & Antonelli, 2013; Verboom, Bergh, Haiden, Hoffmann, & Britton, 2015).

Potential refugia are generally predicted to have a widespread ancestral haplotype, and other mutationally derived and unique haplotypes (Crandall & Templeton, 1993). According to coalescent theory, most ancient haplotypes should be located at the interior nodes of a haplotype network, whereas the most recent haplotypes should be at the tips (Posada & Crandall, 2001). In the cpDNA network of *Triplostegia*, haplotype H2 from SW is at the central position with high frequency (Figure 1). The ancestral haplotype occurs widely across SW, which indicates that the species had persisted over a longer period of time in SW during the glacial epochs. Three populations (SW13, SW14, and SW18) from Muli County, Sichuan Province, have higher haplotype and nucleotide diversity (SW13: *H*_d_ = 0.61, π = 0.34 × 10^−3^; SW14: *H*_d_ = 0.68, π = 0.40 × 10^−3^; SW18: *H*_d_ = 0.67, π = 0.34 × 10^−3^) and contain additional derived and unique haplotypes (Figure 1). Muli County is located in the central Hengduan Mts., with numerous north‐south trending valleys and ridges extending from 1,000 m to over 6,000 m in elevation (Peng, Wan, & Luo, 2000), which might have provided suitable habitats for *Triplostegia* during the Pleistocene climate oscillations. Multiple refugia have been inferred in the Hengduan Mts. for many animals and plants during the Pleistocene glacial periods (Gao et al., 2007; Sun et al., 2014; Wen et al., 2014; Ye et al., 2014; Zhang et al., 2015).

### 4.2 Biogeographic history

Our analyses revealed that coalescence of all *Triplostegia* cpDNA haplotypes is most likely to have occurred in SW, and diversification initiated during the late Miocene (*c*. 6.79 Ma; 95% HPD: 2.50–13.6 Ma; node I in Figures 4 and S5). The late Miocene has been recognized as an important period of diversification for both woody and herbaceous plants (e.g., *Heterobalanus*, Meng et al., 2017; *Cercidiphyllum*, Qi et al., 2012; *Tetracentron*, Sun et al., 2014; *Tetrastigma*, Wang, Jiang et al., 2015). Differentiation within *Triplostegia* might have been initiated and driven by both uplift of the Hengduan Mts. and global cooling after the late Miocene (Nagalingum et al., 2011; Zachos, Dickens, & Zeebe, 2008). The Hengduan Mts., at the southeastern margin of the QTP, are proposed to have experienced rapid uplift during the late Miocene (Clark et al., 2005; Kirby et al., 2002). By investigating the evolutionary histories of multiple plant groups, Xing and Ree (2017) detected an increase in the rate of in situ diversification in the Hengduan Mts. during the late Miocene (*c*. 8.00 Ma). The SW origin of *Triplostegia* is further supported by our phylogenetic reconstruction based on nine complete chloroplast genomes (Niu et al., unpublished) that represented populations from SW, CC, and TW, which strongly supported (PP = 1.00; BS = 100%) initial divergence of the SW clade, and CC and TW populations grouped together (Figure S8). In addition, previous phylogenetic and phylogeographic studies generally inferred that extant populations in TW are descendants from Mainland China (e.g., *Taiwania*, Chou, Thomas, Ge, LePage, & Wang, 2011; *Cunninghamia konishii*, Lu et al., 2001; *Sassafras*, Nie, Wen, & Sun, 2007; *Dichocarpum arisanense*, Xiang et al., 2017), with no reported cases that supported a reverse dispersal direction.

Ancestral area reconstruction revealed that *Triplostegia* might have colonized CC during the Pliocene (*c*. 4.03 Ma; 95% HPD: 1.30–8.60 Ma; node II in Figure 4) as the global temperature continued to decrease. Concurrently, accelerated uplift of the eastern QTP during the late Miocene and Pliocene induced dramatic geomorphological changes in HHM (Favre et al., 2015; Ge et al., 2013; Mulch & Chamberlain, 2006). The absence of shared haplotypes between SW and CC indicates a lack of gene flow between the two regions that was probably due to habitat isolation caused by elevation gradients (average elevation for populations in SW is *c*. 900 m higher than those from CC; Table S1). Morphologically, CC populations also differed from SW populations based on the presence of a serrate leaf blade (vs. pinnatifid leaf blade in SW; Figure 4). Divergence between Mainland China and Taiwan populations in *Triplostegia* (*c*. 2.15 Ma, 95% HPD: 0.50–5.30 Ma; node V in Figure 4) and previously studied woody plants (*c*. 4.04 Ma, *Juglans cathayensis*, Bai, Wang, & Zhang, 2015; *c*. 3.31 Ma, *Taiwania cryptomerioides*, Chou, Thomas, Ge, LePage, & Wang, 2011; *c*. 3.29 Ma, *Taxus wallichiana*, Gao et al., 2007) was congruently inferred to be mid‐Pliocene, which was shortly after Taiwan attained its modern form. The lowest differentiation index between CC and TW (Tables S5 and S6) and the more similar leaf blades (Figure 4) further support the idea that TW populations are descendants of CC.

Our SDM analysis provides a detailed picture of the last glacial cycle (Figure 5a,b) and glimpses of the preceding cycles. Based on the paleoclimate record, temperature in each glacial period was similar (Bloom, 2010). Potential suitable habitat distribution of *Triplostegia* in Mainland China at *c*. 2.15 Ma might be similar to that during the LGM (Figure 5b): *Triplostegia* expanded to the coastal regions of East China and reached TW via land bridges in the Taiwan Strait during the glacial period. Because of repeated glacial–interglacial cycles, CC and TW populations may have experienced frequent gene flow via land bridges during the Quaternary. However, genetic exchange between SW and CC might have been obstructed by altitudinal gradients. This may explain why CC haplotypes are phylogenetically closer to those of distant TW rather than the nearby SW. Nie et al. (2007) and Xiang et al. (2017) also support our scenario that migration existed from CC to TW during the Early Pleistocene by either land bridges or long‐distance dispersal. Neither pollen grains nor seeds of *Triplostegia* have strong dispersal abilities. The long‐distance dispersal hypothesis is thus not likely to be the cause of the disjunct *Triplostegia* distributions. Therefore, current distribution pattern in *Triplostegia* might be the consequence of topographic isolation and postglacial contraction.

### 4.3 Conservation implications

Identifying areas with stable niches and understanding their importance in determining the current distribution of species represent a pivotal task for biodiversity conservation (Marta, Lacasella, Gratton, Cesaroni, & Sbordoni, 2016). The predicted high climatic suitability areas of *Triplostegia* in 2070s are more restricted than that of the present, especially in southern HHM (Figure 5c,d). Additionally, areas with high climatic suitability in Mainland China are predicted to show a slightly northward migration, whereas climatic suitability in Taiwan may suffer from declining (Figure 5d). The potential reason may be that Mainland China has very northern mountain habitats that are suitable for *Triplostegia*; however, Taiwan has neither northern cold areas nor higher mountains for *Triplostegia* dispersal with continued global warming (Figure 5d). Moreover, global mean annual temperatures are likely to rise by an additional 1–4°C by the year 2100 (Stocker et al., 2013). It is undoubtedly a big challenge for the survival of many organisms, especially cold‐adapted taxa that inhabit high mountains, such as *Triplostegia*, which may experience local extinction at the warm edge, and their suitable habitat distribution in the near future will be as limited as or more restricted than during the LIG (Beever, Ray, Wilkening, Brussard, & Mote, 2011; Wiens, 2016).

Species distribution modeling only uses species occurrence data and environmental variables to predict the potential geographical distribution of species (Guisan & Thuiller, 2005). However, climate change is not the sole factor affecting species' habitat suitability. Human activities (e.g., overharvesting, urbanization, fire suppression, and nitrogen pollution) also have profound effects on the distribution of organisms (MacDougall, McCann, Gellner, & Turkington, 2013). Although we predicted a northward range shift under continuing global warming, the potential habitats may be no longer suitable for survival because of human activities. Indeed, we failed to discover *Triplostegia* in numerous places with detailed specimen records. *Triplostegia* represents series of organisms that are already confined to mountaintops (QTP) and/or islands (TW). Therefore, conservation actions are needed to protect these taxa, which are sensitive to climate change and habitat disturbance.

## 5 CONCLUSION

In this study, we explored how geology and climate together influenced the evolutionary history of *Triplostegia*, which is an ecologically sensitive and cold‐adapted herbaceous genus. High genetic divergences were detected among *Triplostegia* populations in SW, CC, and TW, and several interglacial refugia were recognized in the central Hengduan Mts. (particularly Muli County, Sichuan Province). Ancestral area reconstruction revealed that *Triplostegia* originated from SW, and diversification began during the late Miocene as a result of global cooling and uplift of the Hengduan Mts., then colonized CC in the mid‐Pliocene when global temperature further decreased, and finally dispersed to TW by land bridge at *c*. 2.15 Ma. Based on phylogeographic and SDM analyses, frequent gene flow might have existed between CC and TW populations with repeated continued distribution between two regions during the Pleistocene glacial cycles. Nevertheless, biotic exchange between SW and CC might have been isolated by altitudinal gradients triggered by dramatic physiographic changes in HHM. Our research represents the first comprehensive phylogeographic study on herbaceous plants to investigate the HHM‐TW disjunction and supports that postglacial range contraction together with topographic heterogeneity resulted in the HHM‐TW disjunction.

## CONFLICT OF INTEREST

None declared.

## AUTHOR CONTRIBUTIONS

L.‐M.L., J.‐F.Y., and Z.‐D.C. planned and designed the research. Y.‐T.N. performed experiments. Y.‐T.N., J.‐Z.W., and T.Y. analyzed the data. Z.‐D.C., J.‐F.Y., L.‐M.L., and T.Y. conducted the field work. L.‐M.L., Z.‐D.C., Y.‐T.N., J.‐L.Z., X.‐X.W., and J.‐H.L. wrote the manuscript.

## DATA ACCESSIBILITY

All haplotype sequences identified in this study were deposited in GenBank (accession numbers MF737219–MF737340).
