# Supplementary material for: Long‐distance dispersal or postglacial contraction? Insights into disjunction between Himalaya–Hengduan Mountains and Taiwan in a cold‐adapted herbaceous genus, Triplostegia
Source: Ecol Evol. 2017 Dec 20;8(2):1131–46. doi: 10.1002/ece3.3719 (PMC5773327; doi:10.1002/ece3.3719)
Supplement: Supplementary file 1 [file ECE3-8-1131-s001.docx]

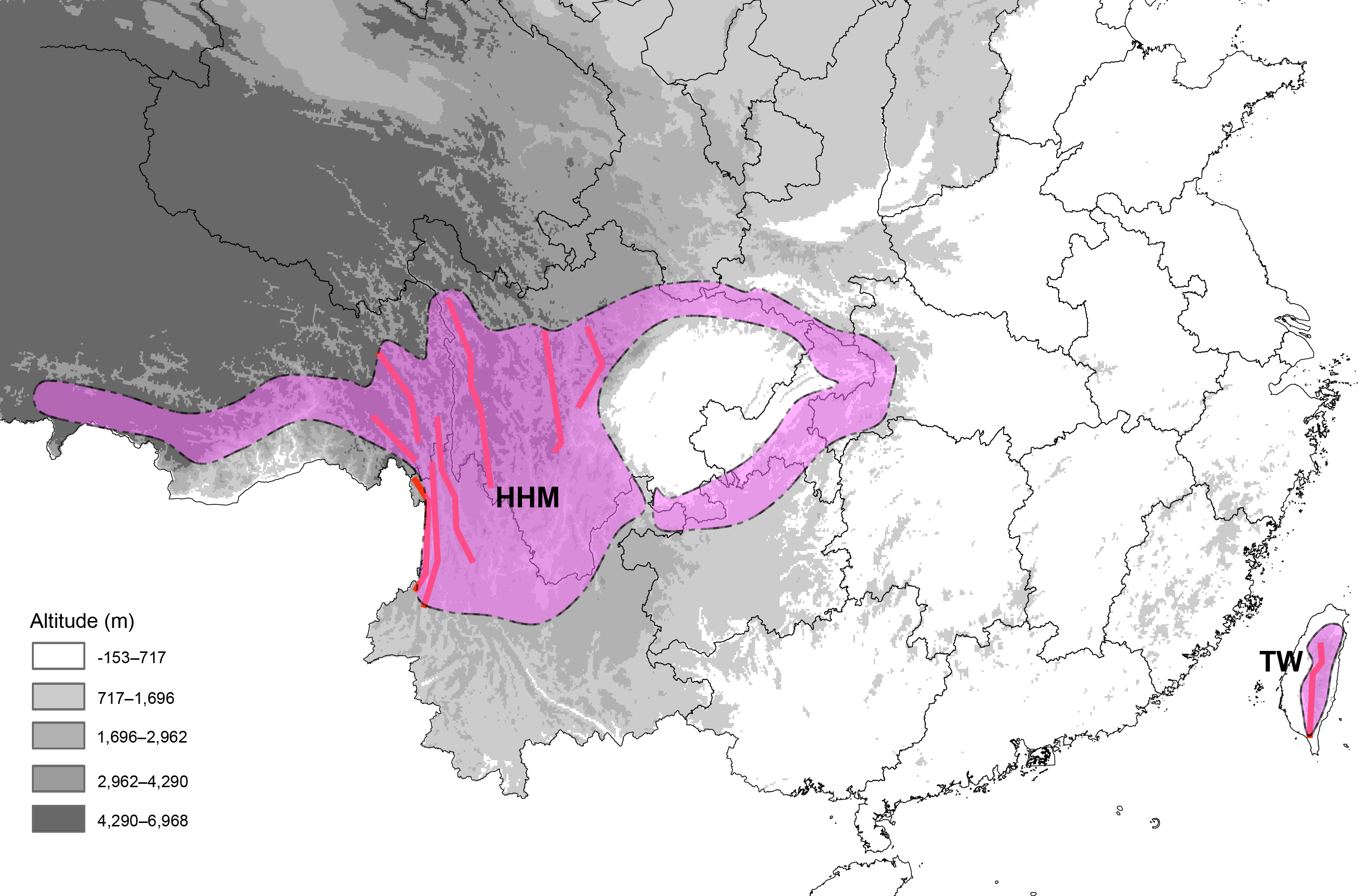


**FIGURE S1** Disjunct distributions of *Triplostegia*. Regions in magenta represent the distribution range of *Triplostegia*; HHM, the Himalaya-Hengduan Mountains and adjacent areas; TW, the Central Mountain range of Taiwan.

**
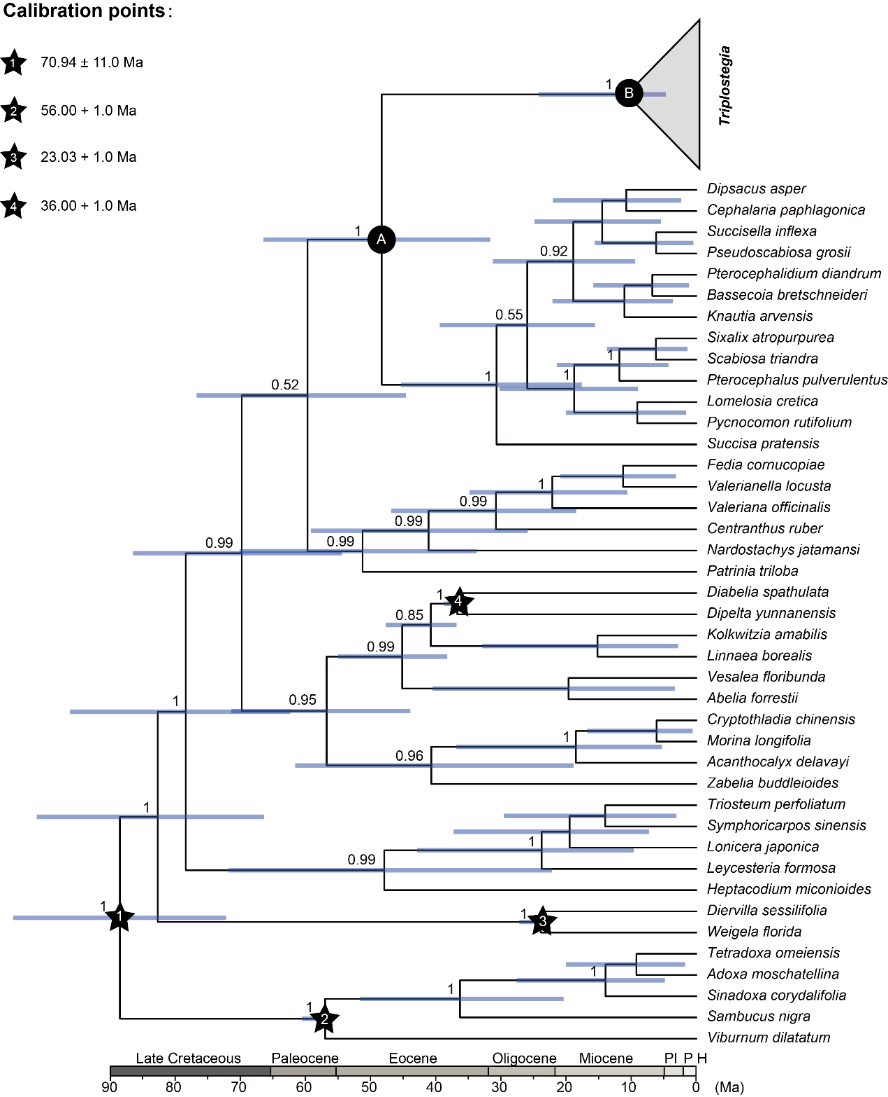
**

**FIGURE S2** BEAST-derived chronogram of Dipsacales based on cpDNA (*trn*L*–*F, *trn*H*–psb*A, and *trn*S*–trn*G) sequences with four calibrations denoted at nodes 1–4 (see Table S7 for details). Blue bars indicate the 95% highest posterior density (HPD) credibility intervals for node ages. Nodes A and B were then used as calibrations to estimate divergence times of all haplotypes derived from the five cpDNA markers. The [grey](javascript:void(0);) [triangle](javascript:void(0);) represents the haplotypes derived from the three cpDNA markers.

**
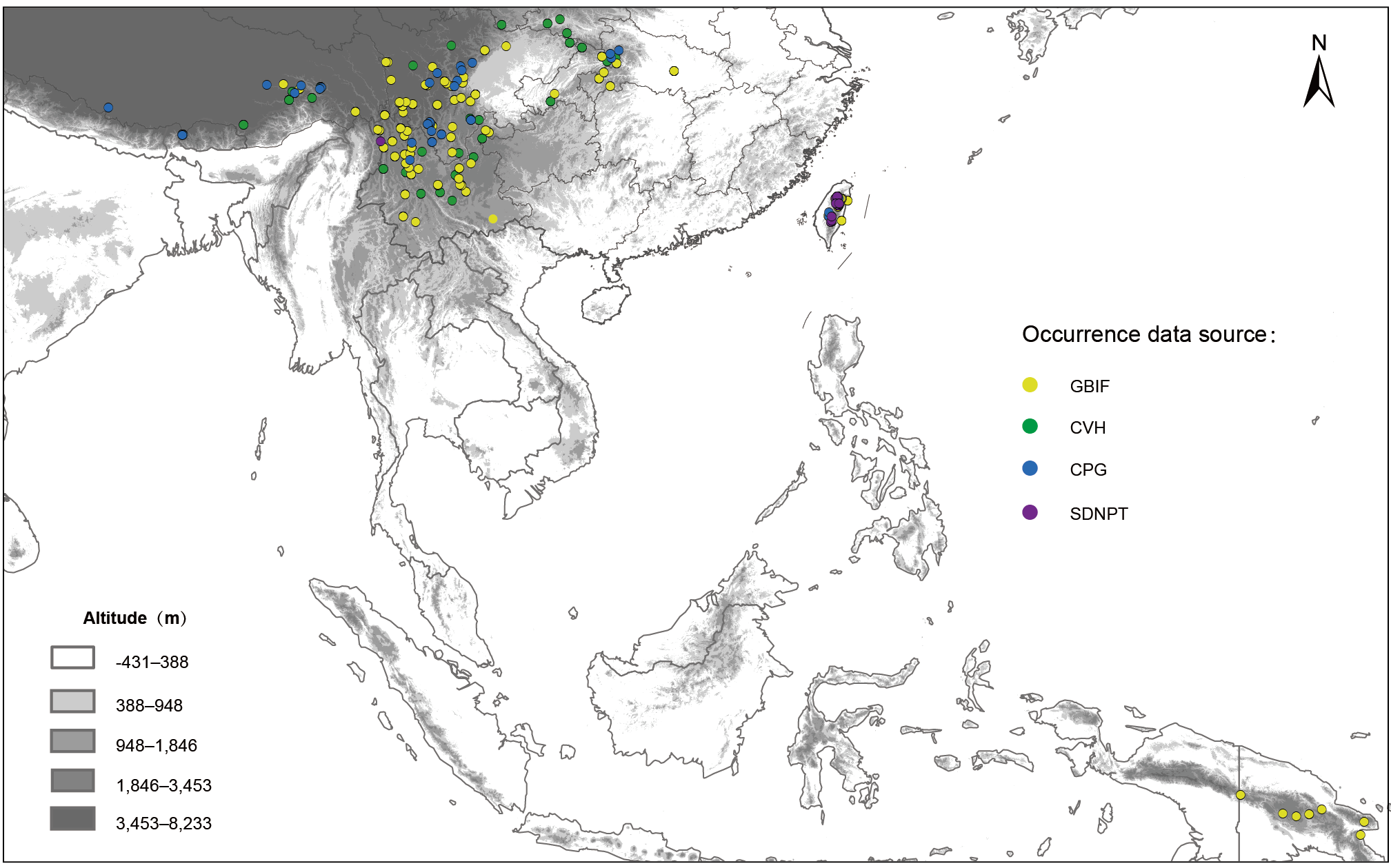
**

**FIGURE S3** Distribution of the cleaned occurrence records of *Triplostegia* from different databases. GBIF: Global Biodiversity Information Facility; CVH: Chinese Virtual Herbarium; CPG: Our collections; SDNPT: Specimens Database of Native Plants in Taiwan.

**

FIGURE S4** Comparison of species distribution predictions under different global climate models (GCMs): (a)–(c), the Last Glacial Maximum (LGM; 0.021 Ma) under three GCMs (CCSM4, MIROC-ESM and MPI-ESM-P); (e)–(f), representative concentration pathways (RCP) 4.5 for 2050s (2041–2060); (g)–(i), RCP 8.5 for 2050s; (j)-(l), RCP 4.5 for 2070s (2061–2080); (m)–(n), RCP 8.5 for 2070s under three GCMs (CCSM4, MIROC-ESM and MPI-ESM-LR). Climatic suitability increases with color from blue to red. Resolution for the potential distribution map is 2.5-arc-minutes.

**
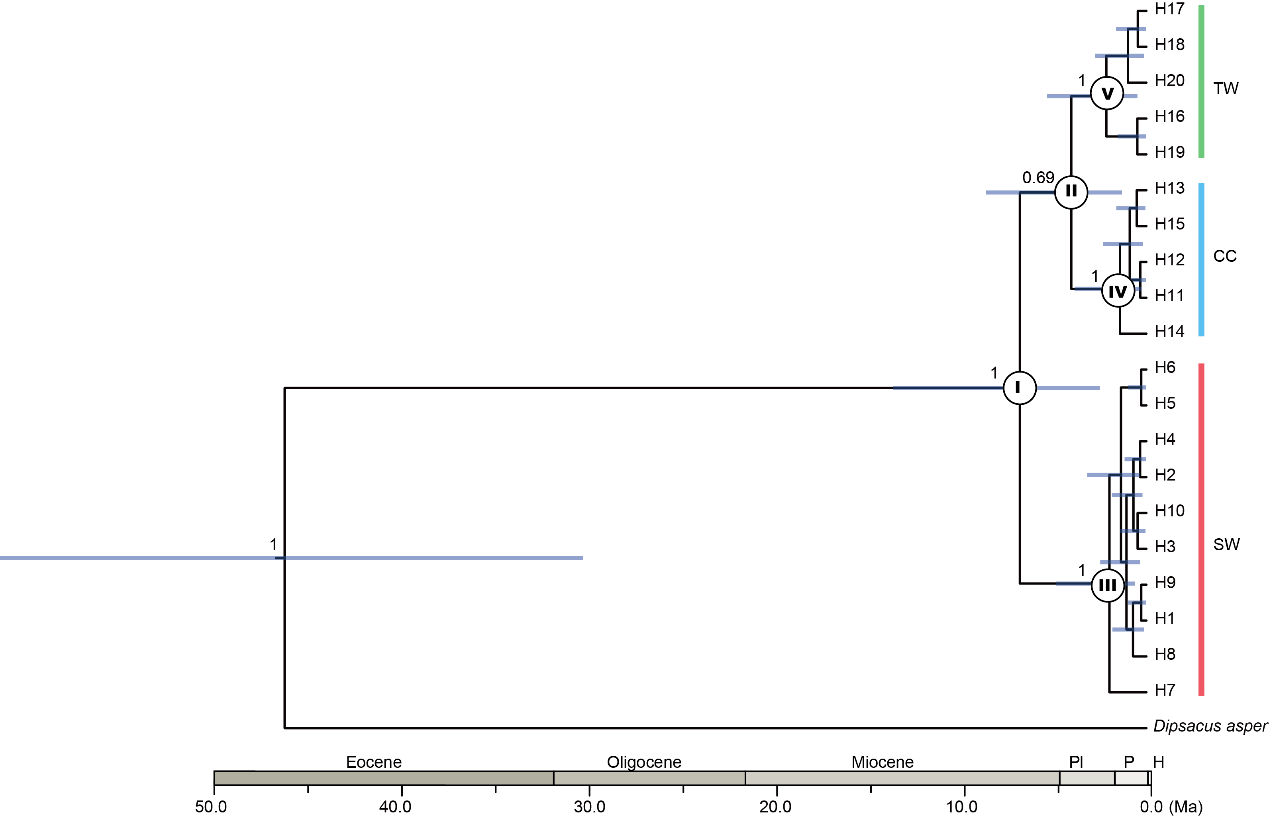
**

**FIGURE S5** Chronogram based on all haplotypes derived from five cpDNA regions. Pl, Pleistocene; P, Pliocene; H, Holocene. Blue bars represent the 95% highest posterior density (HPD) confidence intervals for node ages. Posterior probabilities of key nodes are labeled above branches.

**
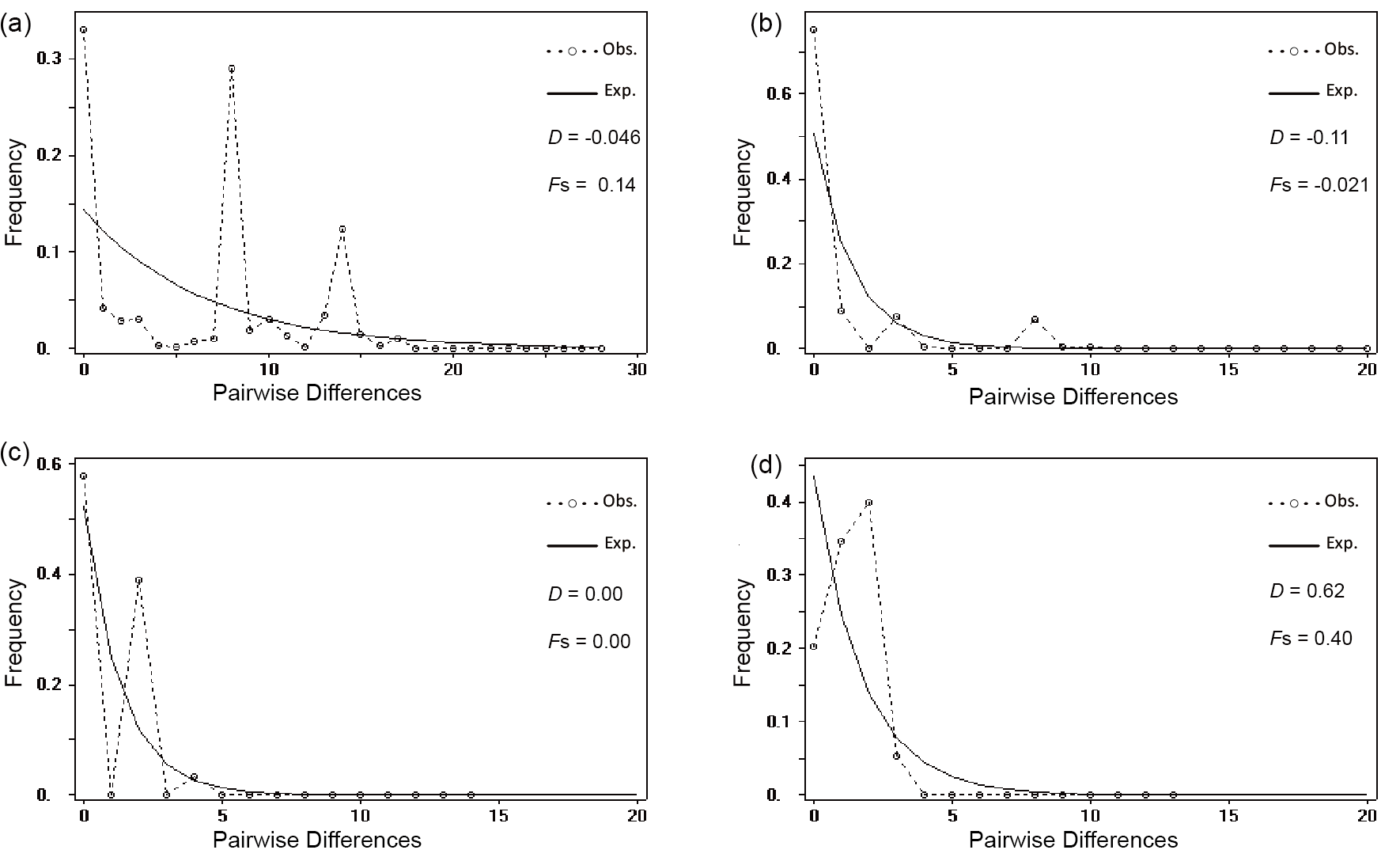
FIGURE S6** Number of pairwise nucleotide differences and neutrality tests in *Triplostegia* based on ITS sequences for (a) all areas, (b) Southwest China, (c) Central China, and (d) Taiwan. White dots and dashed lines show observed values (Obs.); solid lines indicate expected values (Exp.) under a model of sudden (stepwise) population expansion.

**
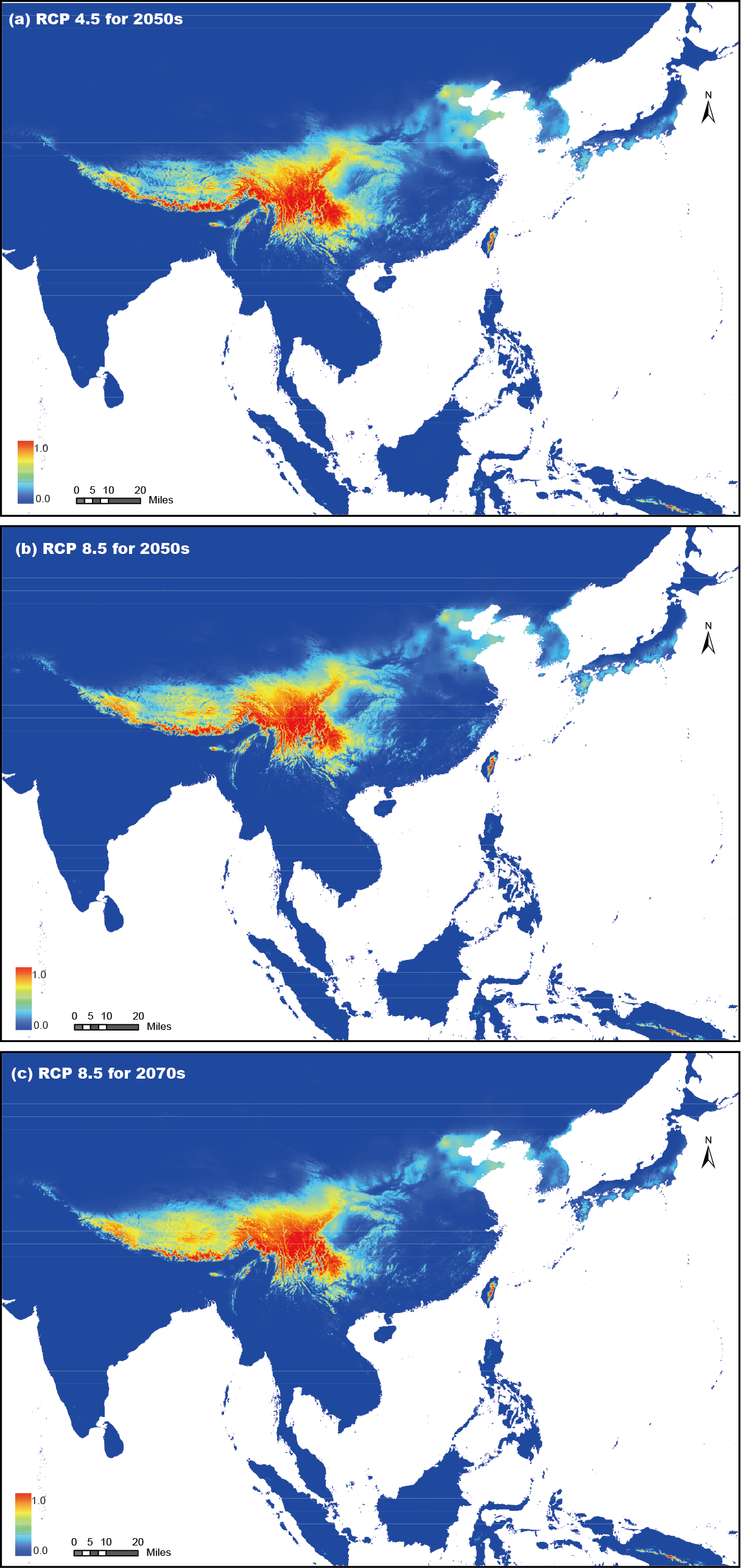
**

**FIGURE S7** Species distribution models showing future climatic suitability for *Triplostegia* in East Asia: (a) representative concentration pathways (RCP) 4.5 for 2050s; (b) RCP 8.5 for 2050s; and (c) RCP 8.5 for 2070s. Climatic suitability increases with color from blue to red. Resolution for the potential distribution map is 2.5 arc-minutes.

**
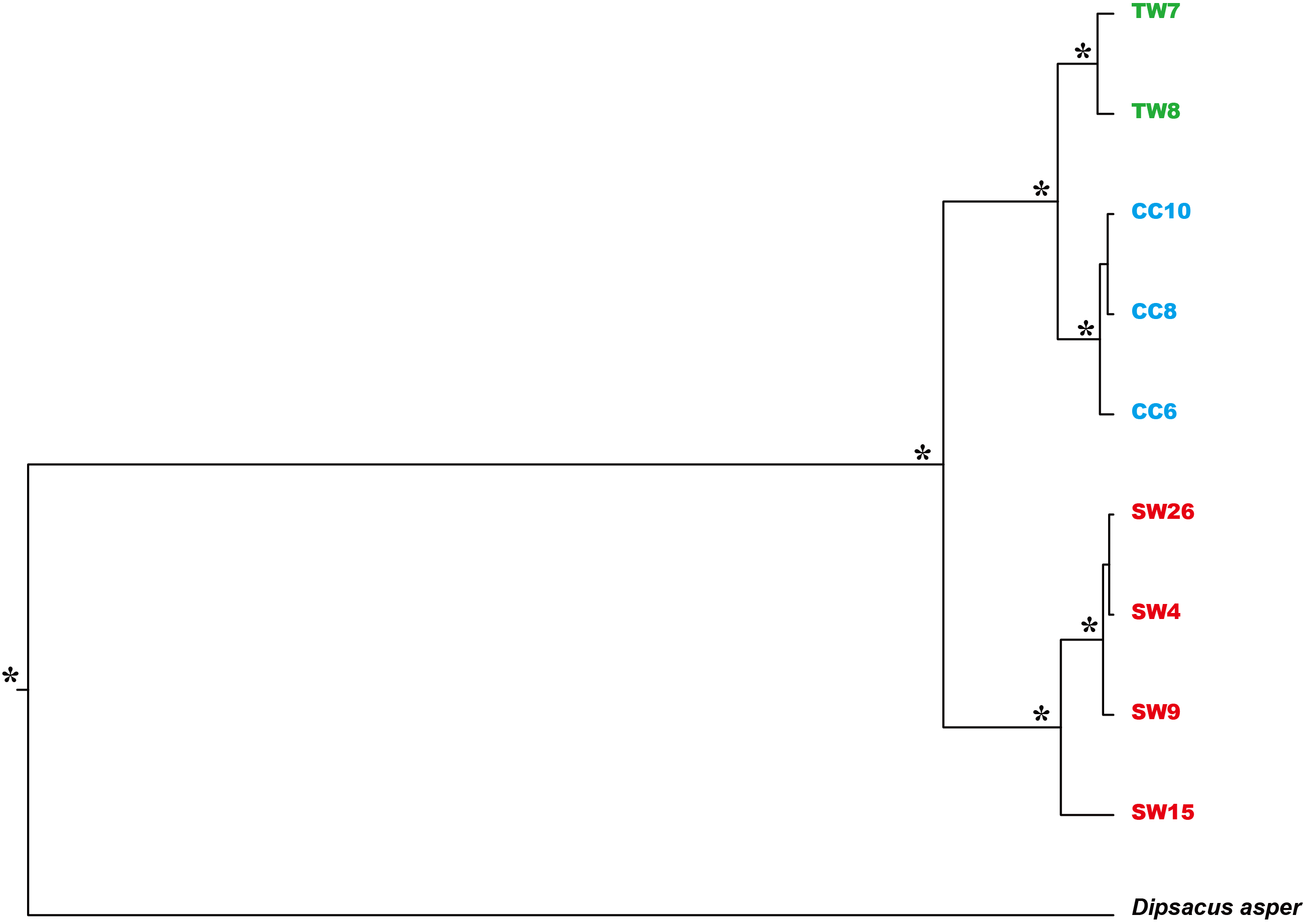
**

**FIGURE S8** Phylogenetic relationship of *Triplostegia* individuals from nine representative populations based on complete chloroplast genomes (Niu *et al*., unpublished). * indicates Bayesian posterior probability of 1.00 and ML bootstrap of 100%.

**TABLE S1** Details of location, size and haplotype information of populations

| **Population** | | **Location** | **Longitude (°E)** | **Latitude (°N)** | **Altitude(m)** | **No. of individuals** | **cpDNA** | | | **nuDNA** | | |
| --- | --- | --- | --- | --- | --- | --- | --- | --- | --- | --- | --- | --- |
|  |  |  |  |  |  |  | **haplotype** | ***H*d** | ***π* (**× 10^-3^) | **haplotype** | ***H*d** | ***π*(**× 10^-3^) |
| SW1 | Heqing, Yunnan | | 100.16 | 26.27 | 2800 | 11 | H1(11) | 0 | 0 | N1(11) | 0 | 0 |
| SW2 | Lijiang, Yunnan | | 100.25 | 27.14 | 3300 | 8 | H2(8) | 0 | 0 | N2(8) | 0 | 0 |
| SW3 | Linzhi, Xizang | | 94.49 | 29.58 | 3200 | 8 | H3(3), H4(5) | 0.54 | 0.38 | N2(8) | 0 | 0 |
| SW4 | Linzhi, Xizang | | 94.80 | 29.95 | 3200 | 7 | H4(7) | 0 | 0 | N2(7) | 0 | 0 |
| SW5 | Bomi, Xizang | | 95.79 | 29.85 | 2660 | 8 | H3(6), H4(2) | 0.43 | 0.31 | N2(8) | 0 | 0 |
| SW6 | Bomi, Xizang | | 95.79 | 29.85 | 2660 | 8 | H3(8) | 0 | 0 | N2(8) | 0 | 0 |
| SW7 | Daofu, Sichuan | | 101.51 | 30.57 | 3520 | 8 | H2(8) | 0 | 0 | N2(8) | 0 | 0 |
| SW8 | Xiaojin, Sichuan | | 102.65 | 30.89 | 3510 | 8 | H2(8) | 0 | 0 | N2(8) | 0 | 0 |
| SW9 | Yanbian, Sichuan | | 101.25 | 27.16 | 2876 | 8 | H2(8) | 0 | 0 | N2(8) | 0 | 0 |
| SW10 | Yanbian, Sichuan | | 101.24 | 27.16 | 2876 | 8 | H2(8) | 0 | 0 | N2(8) | 0 | 0 |
| SW11 | Yanbian, Sichuan | | 101.25 | 27.15 | 2876 | 9 | H2(9) | 0 | 0 | N2(9) | 0 | 0 |
| SW12 | Muli, Sichuan | | 101.18 | 28.08 | 2866 | 8 | H2(7), H5(1) | 0.25 | 0.090 | N2(8) | 0 | 0 |
| SW13 | Muli, Sichuan | | 101.15 | 28.11 | 3261 | 8 | H2(5), H5(1), H6(2) | 0.61 | 0.34 | N2(8) | 0 | 0 |
| SW14 | Muli, Sichuan | | 101.13 | 28.13 | 3261 | 8 | H2(4), H5(1), H6(3) | 0.68 | 0.40 | N2(8) | 0 | 0 |
| SW15 | Muli, Sichuan | | 101.04 | 28.06 | 2400 | 10 | H7(10) | 0 | 0 | N3(10) | 0 | 0 |
| SW16 | Muli, Sichuan | | 101.04 | 28.06 | 2967 | 8 | H2(8) | 0 | 0 | N2(8) | 0 | 0 |
| SW17 | Muli, Sichuan | | 101.04 | 28.06 | 2967 | 8 | H2(2), H8(6) | 0.43 | 0.15 | N2(8) | 0 | 0 |
| SW18 | Muli, Sichuan | | 101.22 | 27.69 | 3234 | 9 | H1(2), H2(2), H4(5) | 0.67 | 0.34 | N2(8), N4(1) | 0.22 | 0.39 |
| SW19 | Yanyuan, Sichuan | | 101.72 | 27.54 | 3149 | 8 | H1(7), H9(1) | 0.25 | 0.12 | N2(7), N4(1) | 0.25 | 0.44 |
| SW20 | Yanyuan, Sichuan | | 101.72 | 27.53 | 3149 | 8 | H1(5), H9(3) | 0.54 | 0.19 | N2(7), N4(1) | 0.25 | 0.44 |
| SW21 | Yadong, Xizang | | 88.98 | 27.52 | 3000 | 8 | H2(8) | 0 | 0 | N2(8) | 0 | 0 |
| SW22 | Yadong, Xizang | | 88.98 | 27.53 | 3100 | 8 | H2(8) | 0 | 0 | N2(8) | 0 | 0 |
| SW23 | Yadong, Xizang | | 88.95 | 27.52 | 3600 | 8 | H2(4), H4(4) | 0.57 | 0.20 | N2(8) | 0 | 0 |
| SW24 | Yadong, Xizang | | 88.94 | 27.52 | 3700 | 8 | H2(4), H4(4) | 0.57 | 0.20 | N2(8) | 0 | 0 |
| SW25 | Yadong, Xizang | | 88.95 | 27.51 | 3800 | 8 | H2(6), H4(2) | 0.43 | 0.15 | N2(8) | 0 | 0 |
| SW26 | Jilong, Xizang | | 85.31 | 28.86 | 3200 | 8 | H2(2), H4(6) | 0.43 | 0.15 | N2(8) | 0 | 0 |
| SW27 | Linzhi, Xizang | | 94.49 | 29.58 | 3200 | 8 | H3(1), H4(7) | 0.25 | 0.18 | N2(8) | 0 | 0 |
| SW28 | Gongbujiangda, Xizang | | 93.11 | 29.98 | 3100 | 8 | H4(6), H8(2) | 0.43 | 0.31 | N2(8) | 0 | 0 |
| SW29 | Bomi, Xizang | | 95.71 | 29.78 | 3700 | 9 | H10(9) | 0 | 0 | N2(9) | 0 | 0 |
| SW30 | Kangding, Sichuan | | 101.13 | 30.07 | 3200 | 10 | H2(10) | 0 | 0 | N5(10) | 0 | 0 |
| **Total (Southwest China)** | | | |  |  | 249 | 10 | 0.72 | 0.38 | 5 | 0.25 | 1.72 |
| CC1 | Tianquan, Sichuan | | 102.50 | 30.19 | 2100 | 10 | H11(10) | 0 | 0 | N6(10) | 0 | 0 |
| CC2 | Baoxing, Sichuan | | 102.72 | 30.69 | 2150 | 7 | H12(7) | 0 | 0 | N7(7) | 0 | 0 |
| CC3 | Leibo, Sichuan | | 103.18 | 28.25 | 2500 | 8 | H13(8) | 0 | 0 | N6(8) | 0 | 0 |
| CC4 | Leibo, Sichuan | | 103.18 | 28.24 | 2500 | 8 | H11(8) | 0 | 0 | N6(8) | 0 | 0 |
| CC5 | Leibo, Sichuan | | 103.17 | 28.25 | 2500 | 6 | H11(6) | 0 | 0 | N6(6) | 0 | 0 |
| CC6 | Leibo, Sichuan | | 103.17 | 28.24 | 2500 | 9 | H11(8), H13(1) | 0.22 | 0.080 | N6(9) | 0 | 0 |
| CC7 | Tianquan, Sichuan | | 102.35 | 29.89 | 1827 | 8 | H14(8) | 0 | 0 | N7(8) | 0 | 0 |
| CC8 | Wenchuan, Sichuan | | 103.23 | 31.08 | 2900 | 10 | H12(5), H15(5) | 0.56 | 0.40 | N6(10) | 0 | 0 |
| CC9 | Wushan, Chongqing | | 110.08 | 31.30 | 1668 | 8 | H12(8) | 0 | 0 | N6(8) | 0 | 0 |
| CC10 | Shennongjia, Hubei | | 110.04 | 31.52 | 1839 | 9 | H12(9) | 0 | 0 | N6(9) | 0 | 0 |
| CC11 | Shennongjia, Hubei | | 110.45 | 31.67 | 2136 | 9 | H12(9) | 0 | 0 | N8(9) | 0 | 0 |
| **Total (Central China)** | | | |  |  | 92 | 5 | 0.70 | 0.33 | 3 | 0.42 | 1.60 |
| TW1 | Nantou, Taiwan | | 121.30 | 24.14 | 3100 | 12 | H16(5), H17(7) | 0.53 | 0.95 | N9(12) | 0 | 0 |
| TW2 | Xueshan, Taiwan | | 121.30 | 24.39 | 3300 | 8 | H18(8) | 0 | 0 | N10(8） | 0 | 0 |
| TW3 | Hehuanxi, Taiwan | | 121.29 | 24.15 | 2600 | 9 | H16(9) | 0 | 0 | N10(6), N11(3) | 0.50 | 0.88 |
| TW4 | Yushan, Taiwan | | 120.90 | 23.48 | 1500 | 8 | H19(7), H20(1) | 0.25 | 0.45 | N12(8) | 0 | 0 |
| TW5 | Yushan, Taiwan | | 120.89 | 23.48 | 2000 | 8 | H20(8) | 0 | 0 | N12(8) | 0 | 0 |
| TW6 | Alishan, Taiwan | | 120.80 | 23.51 | 2600 | 8 | H20(8) | 0 | 0 | N13(8) | 0 | 0 |
| TW7 | Xitou, Taiwan | | 120.80 | 23.69 | 1150 | 3 | H18(3) | 0 | 0 | N14(3) | 0 | 0 |
| **Total (Taiwan)** | | |  |  |  | 56 | 5 | 0.79 | 1.02 | 6 | 0.80 | 2.29 |
| **Total** |  | |  |  |  | 397 | H1～H20 | 0.87 | 2.44 | N1～N14 | 0.67 | 10.58 |

**TABLE S2** Primers and amplification profiles for PCR

| **Region** | **Primer** | **Sequence (5**’ **to 3**’) | **PCR parameters** | **References** |
| --- | --- | --- | --- | --- |
| ITS | ITS1  ITS4 | CCTTATCATTTAGAGGAAGGAG  TCCTCCGCTTATTGATATGC | 5 min at 95 °C, then 37 cycles each of 1 min at 94 °C, 30 s at 50 °C, and 1 min 30 s at 72 °C, followed by a final extension of 10 min at 72 °C. | (White et al., 1990; Baldwin, 1993) |
| *trn*L–F | c  f’ | CGAAATCGGTAGACGCTACG  ATTTTCAGTCCTCTGCTCTACC | 3 min at 95 °C, then 37 cycles each of 20 s at 94 °C, 30 s at 50 °C, and 40 s at 72 °C, followed by a final extension of 5 min at 72 °C. | (Taberlet et al*.*, 1991; Soejima & Wen, 2006) |
| *psb*K–*psb*I | *psb*K  *psb*I | TTAGCCTTTGTTTGGCAAG  AGAGTTTGAGAGTAAGCAT | 3 min at 95 °C, then 37 cycles each of 20 s at 94 °C, 30 s at 50 °C, and 40 s at 72 °C, followed by a final extension of 5 min at 72 °C. | (Lahaye et al*.*, 2008) |
| *trn*H–*psb*A | F  R | GTTATGCATGAACGTAATGCTC  CGCGCATGGTGGATTCACAAATC | 3 min at 95 °C, then 37 cycles each of 20 s at 94 °C, 30 s at 50 °C, and 40 s at 72 °C, followed by a final extension of 5 min at 72 °C. | (Sang et al*.*, 1997) |
| *trn*S–*trn*G | *trn*S  *trn*G | GCCGCTTTAGTCCACTCAGC  GAACGAATCACACTTTTACCAC | 4 min at 94°C, then 36 cycles each of 40s at 94°C, 45s at 55°C, and 1min 15 s at 72 °C, followed by a final extension of 10 min at 72 °C. | (Hamilton, 1999) |
| *rpl*20–*rps*12 | *rpl*20  *rps*12 | TTTGTTCTACGTCTCCGAGC  GTCGAGGAACATGTACTAGG | 4 min at 94°C, then 35 cycles each of 40s at 94°C, 45s at 48°C, and 1min 30 s at 72 °C, followed by a final extension of 10 min at 72 °C. | (Hamilton, 1999) |

**TABLE S3** GenBank accession numbers of newly obtained cpDNA and ITS haplotype sequences for *Triplostegia* in this study

| Chlorotype | | *psb*K–*psb*I | *rpl*20–*rps*12 | *trn*H–*psb*A | *trn*L–F | *trn*S–*trn*G | Ribotype | ITS |
| --- | --- | --- | --- | --- | --- | --- | --- | --- |
| H1 | MF737234 | MF737254 | MF737277 | MF737297 | MF737319 | N1 | MF737219 |  |
| H2 | MF737235 | MF737255 | MF737278 | MF737298 | MF737320 | N2 | MF737220 |  |
| H3 | MF737236 | MF737256 | MF737279 | MF737299 | MF737321 | N3 | MF737221 |  |
| H4 | MF737237 | MF737257 | MF737280 | MF737300 | MF737322 | N4 | MF737222 |  |
| H5 | MF737238 | MF737258 | MF737281 | MF737301 | MF737323 | N5 | MF737223 |  |
| H6 | MF737239 | MF737259 | MF737282 | MF737302 | MF737324 | N6 | MF737224 |  |
| H7 | MF737240 | MF737260 | MF737283 | MF737303 | MF737325 | N7 | MF737225 |  |
| H8 | MF737241 | MF737261 | MF737284 | MF737304 | MF737326 | N8 | MF737226 |  |
| H9 | MF737242 | MF737262 | MF737285 | MF737305 | MF737327 | N9 | MF737227 |  |
| H10 | MF737243 | MF737263 | MF737286 | MF737306 | MF737328 | N10 | MF737228 |  |
| H11 | MF737244 | MF737264 | MF737287 | MF737307 | MF737329 | N11 | MF737229 |  |
| H12 | MF737245 | MF737265 | MF737288 | MF737308 | MF737330 | N12 | MF737230 |  |
| H13 | MF737246 | MF737266 | MF737289 | MF737309 | MF737331 | N13 | MF737231 |  |
| H14 | MF737247 | MF737267 | MF737290 | MF737310 | MF737332 | N14 | MF737232 |  |
| H15 | MF737248 | MF737268 | MF737291 | MF737311 | MF737333 |  |  |  |
| H16 | MF737249 | MF737269 | MF737292 | MF737312 | MF737334 |  |  |  |
| H17 | MF737250 | MF737270 | MF737293 | MF737313 | MF737335 |  |  |  |
| H18 | MF737251 | MF737271 | MF737294 | MF737314 | MF737336 |  |  |  |
| H19 | MF737252 | MF737272 | MF737295 | MF737315 | MF737337 |  |  |  |
| H20 | MF737253 | MF737273 | MF737296 | MF737316 | MF737338 |  |  |  |
| *Dipsacus asper* | MF737233 | MF737274 | MF737276 | MF737318 | MF737340 |  |  |  |
| *Scabiosa triandra* | MF737275 | – | MF737275 | MF737317 | – |  |  |  |

“–” represents missing data.

**TABLE S4** NCBI accession numbers of DNA sequences used to estimate divergence time of Dipsacales

| Species | *trn*L–F | *trn*H–*psb*A | *trn*S–*trn*G |
| --- | --- | --- | --- |

| *Adoxa moschatellina* L. | AF366927 | FJ395478 | EF490255 |
| --- | --- | --- | --- |
| *Sambucus nigra* L. | DQ679816 | HG800569 | HQ714431 |
| *Sinadoxa corydalifolia* C.Y.Wu, Z.L.Wu & R.F.Huang | AF366926 | – | EF490263 |
| *Tetradoxa omeiensis* (H. Hara) C.Y.Wu | – | – | EF490264 |
| *Viburnum dilatatum* Thunb. | JN102151 | AY627392 | HQ591828 |
| *Heptacodium miconioides* Rehder | KP297751 | – | EU265326 |
| *Leycesteria formosa* Wall. | KP297753 | KP297632 | KP297787 |
| *Lonicera japonica* Thunb. | HM228580 | HM228536 | AB937500 |
| *Symphoricarpos sinensis* Rehder | KP297757 | KP297634 | KP297791 |
| *Triosteum perfoliatum* L. | AF265291 | – | EU265335 |
| *Diervilla sessilifolia* Buckley | GU168699 | – | – |
| *Weigela florida* (Bunge) A. DC. | KP297764 | KP297633 | KP297798 |
| *Bassecoia bretschneideri* (Batalin) B.L.Burtt | FJ640647 | – | – |
| *Cephalaria paphlagonica* Bobrov | FJ640659 | – | – |
| *Knautia arvensis* (L.) Coult. | FJ640666 | – | – |
| *Lomelosia cretica* (L.) Greuter & Burdet | FJ640689 | – | – |
| *Pseudoscabiosa grosii* (Font Quer) Devesa | FJ640648 | – | – |
| *Pterocephalidium diandrum* (Lag.) G. López | AJ427382 | – | – |
| *Pterocephalus pulverulentus* Boiss. & Balansa | FJ640678 | – | – |
| *Pycnocomon rutifolium* (Vahl) Hoffmanns. & Link | FJ640686 | – | – |
| *Sixalix atropurpurea* (L.) Greuter & Burdet | FJ640667 | – | – |
| *Succisa pratensis* Moench | AY290007 | – | – |
| *Succisella inflexa* (Kluk) G. Beck | AJ427392 | – | – |
| *Abelia forrestii* (Diels) W.W.Sm. | KP297738 | KP297608 | KP297773 |
| *Diabelia spathulata* (Siebold & Zucc.) Landrein | KP297747 | KP297618 | KP297782 |
| *Dipelta yunnanensis* Franch. | KP297750 | KP297620 | KP297785 |
| *Kolkwitzia amabilis* Graebn. | KP297752 | KP297621 | KP297786 |
| *Linnaea borealis* L. | KP297755 | KP297623 | KP297789 |
| *Vesalea floribunda* M.Martens & Galeotti | KP297760 | KP297627 | KP297794 |
| *Acanthocalyx delavayi* (Franch.) M.J.Cannon | AY290003 | – | – |
| *Cryptothladia chinensis* (P.Y.Pai) M.J.Cannon | AF366925 | – | – |
| *Morina longifolia* Wall. ex DC. | GU168710 | – | – |
| *Centranthus ruber* (L.) DC. | AF446986 | AY794225 | – |
| *Fedia cornucopiae* (L.) Gaertn. | JF269260 | AY794226 | – |
| *Nardostachys jatamansi* DC. | AF446980 | AY794227 | – |
| *Patrinia triloba* Miq. | KM358536 | AY794228 | – |
| *Valeriana officinalis* L. | AY360120 | AY794273 | – |
| *Valerianella locusta* (L.) Laterr. | DQ354201 | AY794309 | – |
| *Zabelia buddleioides* (W.W.Sm.) Hisauti & H.Hara | KP297765 | KP297630 | KP297799 |

“–” represents missing data.

**TABLE S5** Molecular variances of chloroplast genotypes

| Group | Source of variation | d.f. | SS | VC | PV | Fixation index |
| --- | --- | --- | --- | --- | --- | --- |
| Southwest vs. Central | Among regions | 1 | 696.04 | 5.14 | 87.17 | *F*_CT_ = 0.87^***^ |
|  | Among populations | 39 | 196.95 | 0.59 | 9.96 | *F*_SC_ = 0.78^***^ |
|  | Within populations | 300 | 50.79 | 0.17 | 2.87 | *F*_ST_ = 0.97^***^ |
|  | Total | 340 | 943.78 | 5.90 |  |  |
| Southwest vs. Taiwan | Among regions | 1 | 639.53 | 6.92 | 87.62 | *F*_CT_ = 0.88^***^ |
|  | Among populations | 35 | 217.61 | 0.73 | 9.18 | *F*_SC_ = 0.74^***^ |
|  | Within populations | 268 | 67.90 | 0.25 | 3.21 | *F*_ST_ = 0.97^***^ |
|  | Total | 304 | 925.04 | 7.90 |  |  |
| Central vs. Taiwan | Among regions | 1 | 210.55 | 2.88 | 68.14 | *F*_CT_ = 0.68^***^ |
|  | Among populations | 16 | 146.34 | 1.09 | 25.70 | *F*_SC_ = 0.81^***^ |
|  | Within populations | 130 | 33.89 | 0.26 | 6.16 | *F*_ST_ = 0.94^***^ |
|  | Total | 147 | 390.68 | 4.23 |  |  |

d.f., degree of freedom；SS, sum of squares; VC, variance components; PV, percentage of variation; ^***^, *P* < 0.001, 1000 permutations.

**TABLE S6** Molecular variances of ITS genotypes

| Group | Source of variation | d.f. | SS | VC | PV | Fixation index |
| --- | --- | --- | --- | --- | --- | --- |
| Southwest vs. Central | Among regions | 1 | 572.86 | 4.22 | 86.89 | *F*_CT_ = 0.87^***^ |
|  | Among populations | 39 | 204.02 | 0.63 | 12.93 | *F*_SC_ = 0.99^***^ |
|  | Within populations | 300 | 2.64 | 0.0088 | 0.18 | *F*_ST_ = 0.99^***^ |
|  | Total | 340 | 779.51 | 4.86 |  |  |
| Southwest vs. Taiwan | Among regions | 1 | 641.33 | 6.95 | 91.14 | *F*_CT_ = 0.91^***^ |
|  | Among populations | 35 | 189.09 | 0.65 | 8.59 | *F*_SC_ = 0.97^***^ |
|  | Within populations | 268 | 5.56 | 0.021 | 0.27 | *F*_ST_ = 0.99^***^ |
|  | Total | 304 | 835.97 | 7.63 |  |  |
| Central vs. Taiwan | Among regions | 1 | 230.65 | 3.24 | 83.53 | *F*_CT_ = 0.84^***^ |
|  | Among populations | 16 | 80.70 | 0.62 | 15.89 | *F*_SC_ = 0.96^***^ |
|  | Within populations | 130 | 2.92 | 0.022 | 0.58 | *F*_ST_ = 0.94^***^ |
|  | Total | 147 | 314.27 | 3.87 |  |  |

d.f., degree of freedom；SS, sum of squares; VC, variance components; PV, percentage of variation; ^***^, *P* < 0.001, 1000 permutations.

**TABLE S7** A summary of divergence time estimation results under a Bayesian approach for Dipsacales based on three cpDNA regions (*trn*H–*psb*A, *trn*L–F and *trn*S–*trn*G)

| Node | Type | Mean ages (95% highest posterior density; Ma) | Calibration age (Ma) | Reference |
| --- | --- | --- | --- | --- |
| Node 1*  (Crown age of Dipsacales) | Secondary | 88.47 (72.18–104.81) | 70.94 ± 11.0 | (Magallón *et al*., 2015) |
| Node 2*  (Crown age of Adoxaceae) | Macrofossil | 56.97 (56.03–60.54) | 56.00 + 1.0 | (Wing *et al*., 1995; Baskin *et al*., 2006) |
| Node 3*  (Stem age of *Weigela*) | Seeds | 23.88 (23.03–27.14) | 23.03 + 1.0 | (Dorofeev, 1963) |
| Node4*  (Stem age of *Dipelta*) | Fruits | 36.70 (36.04–38.72) | 36.00 + 1.0 | (Manchester & Donoghue, 1995) |
| Node A  (Stem age of *Triplostegia*) |  | 48.29 (31.65–66.45) |  |  |
| Node B  (Crown age of *Triplostegia*) |  | 10.92 (4.28–23.76) |  |  |

**REFERENCES:**

Baldwin, B. G. (1993). Molecular phylogenetics of *Calycadenia* (Compositae) based on ITS sequences of nuclear ribosomal DNA: chromosomal and morphological evolution reexamined. *American Journal of Botany*, *80*, 222–238.

Baskin, J. M., Hidayati, S. N., Baskin, C. C., Walck, J. L., Huang, Z. Y., & Chien, C. T. (2006). Evolutionary considerations of the presence of both morphophysiological and physiological seed dormancy in the highly advanced euasterids II order Dipsacales. *Seed Science Research*, *16*, 233–242.

Dorofeev, P. (1963) *Tertiary floras of western Siberia*. Moscow-Leningrad: Akademia Nauk SSSR. (In Russian)

Hamilton, M. B. (1999). Four primer pairs for the amplification of chloroplast intergenic regions with intraspecific variation. *Molecular Ecology*, *8*, 521–523.

Lahaye, R., Savolainen, V., Duthoit, S., Maurin, O., & Van der Bank, M. (2008). *A test of psbK–psbI and atpF–atpH as potential plant DNA barcodes using the flora of the Kruger National Park as a model system (South Africa)*. [Nature Precedings document] URL http://precedings.nature.com/documents/1896/version/1. [accessed 16 May 2008].

Magallón, S., Gómez-Acevedo, S., Sánchez-Reyes, L. L., & Hernández-Hernández,T. (2015). A metacalibrated time-tree documents the early rise of flowering plant phylogenetic diversity. *New Phytologist*, *207*, 437–453.

Manchester, S. R., & Donoghue, M. J. (1995). Winged fruits of Linnaeeae (Caprifoliaceae) in the Tertiary of Western North America: *Diplodipelta* gen. nov. *International Journal of Plant Sciences*, *156*, 709–722.

Sang, T., Crawford, D. J., & Stuessy, T. F. (1997). Chloroplast DNA phylogeny, reticulate evolution, and biogeography of *Paeonia* (Paeoniaceae). *American Journal of Botany*, *84*, 1120–1120.

Soejima, A., & Wen, J. (2006). Phylogenetic analysis of the grape family (Vitaceae) based on three chloroplast markers. *American Journal of Botany*, *93*, 278–287.

Taberlet, P., Gielly, L., Pautou, G., & Bouvet, J. (1991). Universal primers for amplification of three non-coding regions of chloroplast DNA. *Plant Molecular Biology*, *17*, 1105–1109.

White, T. J., Bruns, T., Lee, S., & Taylor, J. W. (1990). Amplification and direct sequencing of fungal ribosomal RNA genes for phylogenetics. In: Innis MA, Gelfand DH, Sninsky JJ, White TJ, eds. *PCR protocols: a guide to methods and applications*. San Diego, USA: Academic Press, 315–322.

Wing, S. L., Alroy, J., & Hickey, L. J. (1995). Plant and mammal diversity in the Paleocene to early Eocene of the Bighorn Basin. *Palaeogeography, Palaeoclimatology, Palaeoecology*, *115*, 117–155.
